# Evaluation of an AI-Based Constraint-Optimization Scheduler to Optimize On-Call Schedule Equity and Reduce Administrative Burden in a Pediatric Residency: Retrospective Comparative Study

**DOI:** 10.2196/88340

**Published:** 2026-07-31

**Authors:** David Gilad, Tzofnat Farbstein-Aljanati, Reut Kassif Lerner, Moshe Ashkenazi, Itai M Pessach

**Affiliations:** 1 Edomand and Lily Safra Children's Hospital, Sheba Medical Center, Emek Dotan St, Ramat Gan, Ramat Gan, Tel Aviv, Israel, 972 35302895

**Keywords:** artificial intelligence, AI-based scheduling, constraint optimization, pediatric residency, scheduling fairness, healthcare operations, real-world implementation

## Abstract

**Background:**

Resident scheduling is a high-dimensional optimization problem with implications for workload, fatigue risk, and equity. Real-world evaluations of AI-based constraint-optimization in health care are limited.

**Objective:**

This study aimed to evaluate an AI-based constraint-optimization scheduler versus a legacy rule-based scheduler for pediatric residency night calls.

**Methods:**

This is a single-center retrospective before-after study at a 235-bed tertiary pediatric center. Twenty-four consecutive months of night-call rosters were analyzed: preimplementation (January to December 2024, legacy rule-based autoscheduler) and postimplementation (January to December 2025, AI-based constraint-programming scheduler combining a local-search metaheuristic solver with human-in-the-loop review). The analytic unit was the resident-month. Outcomes were workload distribution, threshold exceedances (>6 total and >2 weekend calls/month), undesirable sequences (consecutive weekend calls; call-rest-call; call-rest-call-rest-call), equity (mean absolute error from equal share [MAE-ES], root mean square error from equal share), publication lead time, as well as pre- and postsurvey experience.

**Results:**

We analyzed 6519 shifts across 1530 resident-months (legacy: 803 resident-months/107 physicians; AI: 727/87; weekend share 28.8% [934/3246] vs 28.6% [935/3273]; service mix *P*=.99). Mean calls/resident-month did not decline (4.04 vs 4.50; *P*<.001), but within-period SD was approximately halved. Threshold exceedances fell from 133/803 (16.6%) to 28/727 (3.9%) for >6 calls per month (risk ratio [RR] 0.24, 95% CI 0.15‐0.34) and 89/803 (11.1%) to 21/727 (2.9%) for >2 weekend calls (RR 0.27, 95% CI 0.16‐0.40; both *P*<.001). Undesirable sequences declined: consecutive weekends 24.4→18.7/100 resident-months (RR 0.77; *P*=.02); call-rest-call 51.2→23.4 (RR 0.46); call-rest-call-rest-call 5.6→1.0 (RR 0.18; both *P*<.001). Equity improved overall and within every qualification stratum: MAE-ES −0.26 shifts (95% CI −0.28 to −0.23) and RMSE-ES −0.29 (95% CI −0.32 to −0.26); Senior, Advanced, and Novice strata were all *P*<.001 after Holm correction. Publication lead time more than doubled (10.7→21.2 d; *Δ*+10.5, Cohen *d=*4.78; Cliff *δ*=1.00; *P*<.001). Interrupted time-series confirmed immediate level shifts for >6-call exceedances (β=−8.88; *P*=.004), MAE-ES (β=−0.18; *P*<.001), call-rest-call (β=−13.17; *P*=.002), call-rest-call-rest-call (β=−2.35; *P*=.03), and >2 weekend exceedances (β=−7.32; *P*<.001), with stable postimplementation fairness slopes. Among survey respondents (n=47 pre; n=38 post), software satisfaction rose 6.77→8.71/10, perceived timeliness 3.28→4.61/5, perceived consecutive-night frequency 3.15→4.24, and perceived equity 2.98→3.61 (all *P*≤.006).

**Conclusions:**

An AI-based constraint-optimization scheduler was associated with significantly more equitable on-call workload across all qualification strata, large reductions in high-risk shift sequences and threshold exceedances, and a doubling of publication lead time, despite no reduction in mean per-physician burden once all physicians were retained. Multisite prospective replication is warranted before generalization.

## Introduction

The rising complexity of hospital operations, coupled with persistent administrative burden, has sharpened the need for digital transformation in clinical workforce management [1-3]. For large organizations, health care scheduling exemplifies a high-dimensional optimization problem that is unrealistic to brute force compute (nondeterministic polynomial time [NP]-hard) with increasing problem size [4], particularly for residency programs [5-15]. Scheduling must reconcile regulatory limits and service coverage with evolving trainee competencies, personal constraints, and frequent last-minute changes—pressures that often yield fragile rosters, inequitable workload distribution, and contribute to provider burnout. Despite the high stakes, many health care services still rely on rule-based scheduling solutions or even manual methods [16]. Over the past five years, there have been significant advances in applying AI, machine learning, and optimization techniques to health care scheduling. Modern systems increasingly leverage constraint programming and mixed-integer optimization augmented by AI-driven strategies including heuristics, evolutionary algorithms, and learning-based models [17-19]. Some solutions have begun to combine machine learning with classical optimization by learning soft constraints or preferences from historical schedules via machine learning and then using constraint solvers to generate feasible, high-quality rosters that honor those learned patterns. While these innovations show great promise, their real-world impact in health care remains to be fully validated [17]. Comparable real-world evidence in physician residency programs, however, remains scarce; the few published evaluations are largely qualitative, single-system, and short-horizon [5,6,11,13-15,18,19].

To address this gap, we conducted a retrospective comparative evaluation of an AI-based constraint-optimization scheduler versus a legacy rule-based scheduler in a tertiary pediatric center [20], comparing workload, undesirable patterns, fairness, and publication lead time, and assessing perceptions via resident surveys to evaluate these tools within a real health care setting. Our legacy solution represents a legacy rule-based scheduling application with limited automation and flexibility and required significant manual oversight by chief residents. We hypothesized that explicitly encoded fairness rules and sequence-avoidance constraints would reduce workload exceedances and undesirable patterns, improve fairness, and enable earlier publication compared with a legacy rule-based scheduler.

## Methods

### Study Design and Reporting Framework

We conducted a single-center retrospective before-after comparative study with interrupted time-series (ITS) analysis. The preimplementation period spanned January 1 through December 31, 2024 (12 months) under a legacy rule-based autoscheduler, and the postimplementation period spanned January 1 through December 31, 2025 (12 months) under an AI-based constraint-programming scheduler. This paper builds on a previously published implementation pilot of the AI-based scheduler at the same institution [20].

#### Study Settings

The study was set in the Edmond and Lily Safra Children’s Hospital at Sheba Medical Center. At 235 beds, it is one of the largest tertiary pediatric hospitals in Israel and contains one of the largest pediatric residency programs in Israel with over 70 residents. Residents in Safra carry out approximately 1500 shifts a month, including 9 night calls each day covering 9 distinct services: pediatric intensive care unit, pediatric cardiac intensive care unit, neonatal intensive care unit positions 1 and 2, senior emergency department and junior emergency department, pediatric department A (ped A), pediatric department B (ped B), and hematology and oncology. All night-call shifts in this study therefore correspond to 24‐26 hours of continuous duty. Our residency program presents a large-scale scheduling problem where chief residents are required to meet service demands, manage personal requests, the residency block schedule, and plan residency training and qualifications. All physicians appearing on final executed night-call rosters were retained, including residents, fellows, and stand-in attendings; no minimum monthly shift threshold was applied. The smaller number of distinct on-call physicians in the AI-based era reflected routine year-to-year variation in residency cohort composition and a reduced need for occasional stand-in physicians, while the core on-call staff and total clinical demand remained essentially unchanged.

#### Scheduling Software

The legacy scheduler was a commercial rule-based autoscheduler offering per-shift role designation, time-off and leave management, and a minimum and maximum contract rule for monthly weekday and weekend shift counts, with limited pattern detection. In effect, the legacy tool implemented a fixed sequence of priority heuristics that filled shifts one at a time, with chief residents using spreadsheet pivot tables to detect and manually correct workload imbalance after the autogenerated draft. The postimplementation scheduler is a constraint-programming platform built atop the open-source constraint solver and adapted for health care workforce scheduling. Unlike a rule-based approach, the constraint solver represents the entire monthly assignment problem declaratively and searches the feasible-assignment space using a constraint-based local-search metaheuristic to minimize a composite objective covering coverage, fairness, undesirable-sequence avoidance, and resident-level preferences. Chief residents programmed both hard and soft constraints into the solver, including individual resident skillsets and per block-rotation eligibility, qualification-preferred shift assignments, shift templates with minimum and maximum-demand and assignment-priority rules, complex shift-balance rulesets that compare residents to historical workload, contractual minimum and maximum shift counts, predefined wanted or unwanted shift patterns, and preferred or unpreferred resident pairings. A grouped inventory of constraints implemented by the AI-based scheduler is provided in Multimedia Appendix 1, Section 1. Both platforms published final rosters via web and mobile apps. The schedules compared in this study are the final executed rosters in each era; they incorporate postsolver but prepublication chief-resident review and any postpublication adjustments. Therefore, our comparison evaluates the real-world implementation of each platform with a human in the loop, not the raw solver output of either system. Both schedulers operated in a human-in-the-loop workflow in which chief residents reviewed the autogenerated draft, adjusted assignments to handle qualification edge cases, and integrated postpublication shift swaps. Under the AI-based scheduler, chief residents used the platform’s dynamic statistics dashboards, schedule-simulation feature, and AI-prioritized substitution recommendations to evaluate proposed edits. Logs of these human-made changes were not available for analysis. Because of the integral role of the chief-resident reviewer in both eras, the results we report are best understood as differences between two distinct AI-assisted human scheduling systems rather than between two raw solver approaches.

#### Outcomes

The primary analytic unit was the resident-month, defined as one physician’s complete set of night-call shifts within a single calendar month identified by shift start date. Outcomes were grouped into four families that mirror the Results subheadings: workload distribution (mean and SD of total calls and weekend calls per resident-month, plus the proportion of monthly resident-months exceeding the program-defined thresholds of >6 total calls and >2 weekend calls); undesirable shift sequences (consecutive weekend calls, call-rest-call, and call-rest-call-rest-call patterns; expressed as events per 100 resident-months); schedule equity (mean absolute error from equal share [MAE-ES] and root mean square error from equal share [RMSE-ES], both overall and stratified by qualification cluster; lower values indicate more equitable distribution); and administrative burden (schedule publication lead time, defined as days from publication timestamp to the first day of the upcoming scheduling period). Resident-reported experience was captured separately as a secondary outcome through anonymous pre- and postimplementation surveys.

#### Qualification Strata and Skill Mix

For a comparison of fairness between qualification strata, we inferred a resident’s monthly qualification as the highest level the resident had achieved by the end of that month, determined by the repertoire of shifts completed up to that time. Resident-months were classified as either (1) Senior: pediatric intensive care unit call, pediatric cardiac intensive care unit call, senior emergency department call, (2) Advanced: Neonatal intensive care unit 1‐2, junior emergency department call - without meeting criteria for senior-level shifts) and (3) Novice: pediatric department A and B calls, and hemato-oncology - without meeting criteria for Advanced or Senior. This inference was recomputed monthly per resident using their chronological call history, enabling dynamic progression between strata. These strata were selected based on the order of training progression in our residency program.

#### Publication Lead Time Assessment

To calculate the lead time before publication of the next scheduling period, we extracted the monthly schedule publication times for both the legacy and AI-based software system logs. The dates were parsed using a Python script to calculate the lead time from the publication date to the first day of the published scheduling period, compute the descriptive statistics, perform statistical analysis, and plot the results. The lead time analysis of the January 2024 and January 2025 schedules was not included in this analysis because each was published in the trailing weeks of the prior calendar month; before the study window opened (January 2024) and before the AI-based scheduler entered operational use (January 2025), so neither cycle is attributable to the scheduler under evaluation for its publication month.

#### Resident Questionnaire

Two anonymous cross-sectional surveys were administered via Google Forms to all active residents in the program: the first during the legacy scheduling era (December 14‐15, 2024) and the second after approximately 8 months of AI-based scheduler use (August 22‐27, 2025). Residents had not been informed of the upcoming scheduler change at the time of the legacy survey. Submissions were voluntary and anonymous. The instrument was developed by the residency leadership for this study; it captures ordinal Likert-type ratings of scheduler-relevant experience domains. Negatively framed items (frequency of consecutive nights; schedule lead-time satisfaction) were reverse-scored so that higher values uniformly indicate better experience. The full instrument and reverse-scoring key are provided in Section 3 in Multimedia Appendix 1, Section 3. Of approximately 75 active residents invited in December 2024, 47 responded (response rate 63%); of approximately 80 invited in August 2025, 38 responded (response rate 48%). The denominators are approximate because cross-coverage residents joining mid-month were variably included in the eligible-resident roster at the time of survey distribution; the potential for nonresponse bias is addressed in the Limitations. Responses were anonymous, precluding paired analysis; ordinal responses were compared between eras using two-sided Mann-Whitney *U* tests, and effect sizes were summarized as AI-minus-legacy mean differences with 95% Welch unequal-variance CI.

### Statistical Analysis

Continuous resident-month outcomes were compared with the Welch *t* test and the Mann-Whitney *U* test, with Cohen *d* as the effect size. Threshold exceedance proportions and the cross-tabulated qualification mix were compared using Pearson *χ*^2^ with Yates continuity correction and Fisher exact tests, with risk ratios (RRs) summarized using bootstrap log-RR 95% CIs (5000 iterations). Rates of undesirable shift sequences (events per 100 resident-months) were compared using exact-conditional Poisson rate-ratio tests and Pearson *χ*^2^. For the primary equity outcomes (MAE-ES, RMSE-ES), we used two-sided stratified label-permutation tests (25,000 iterations within mo-of-y× qualification strata, yielding 36 strata in the cluster-stratified analysis) that preserve the empirical distribution of calls under the null of era exchangeability; bootstrap 95% CIs were computed from 5000 stratified resamples, and Holm correction was applied across the three clusters per outcome. The two-sided significance threshold was *α*=.05. We assessed cohort imbalance (803 vs 727 resident-months) by comparing mean and bootstrap-CI estimates that are insensitive to denominator size, and by reporting effect sizes (Cohen *d*, RR, Δ MAE-ES) alongside null-hypothesis tests. To control for preimplementation trends, we performed a segmented-regression ITS analysis on a single 24-month series [21] (Time_Month 1‐12=legacy, 13‐24=AI; intervention boundary between mo 12 and 13). The model was outcome = β₀ + β₁·Time + β₂·Intervention + β₃·Post_Time. The primary estimand was β₂ (immediate level change at implementation); the secondary estimand was the average postperiod effect β₂+6.5·β₃ (postperiod midpoint at mo 6.5 of the 12-mo post-window), with standard errors derived from the full coefficient covariance matrix. Inference used Newey-West heteroscedasticity- and autocorrelation-consistent standard errors with lag 2 [22]; sensitivity at lags 1 and 3 is reported in Section 2 in Multimedia Appendix 1. Analyses were conducted in Python 3.11 (Python Software Foundation) with pandas, numpy, scipy.stats, statsmodels, matplotlib, seaborn, and openpyxl. The complete analysis pipeline is described in Section 2 in Multimedia Appendix 1.

### Ethical Considerations

This study used deidentified operational scheduling data and anonymous resident surveys, with no patient-level data and no identifiable individual images. The Sheba institutional research ethics process determined that the study was exempt from Institutional Review Board review under the secondary-use-of-operational-data provision. Resident schedules used for the analyses were deidentified at extraction and survey responses were anonymous. No participant compensation was provided.

## Results

### Cohort and Resident-Months

**Figure 1.**
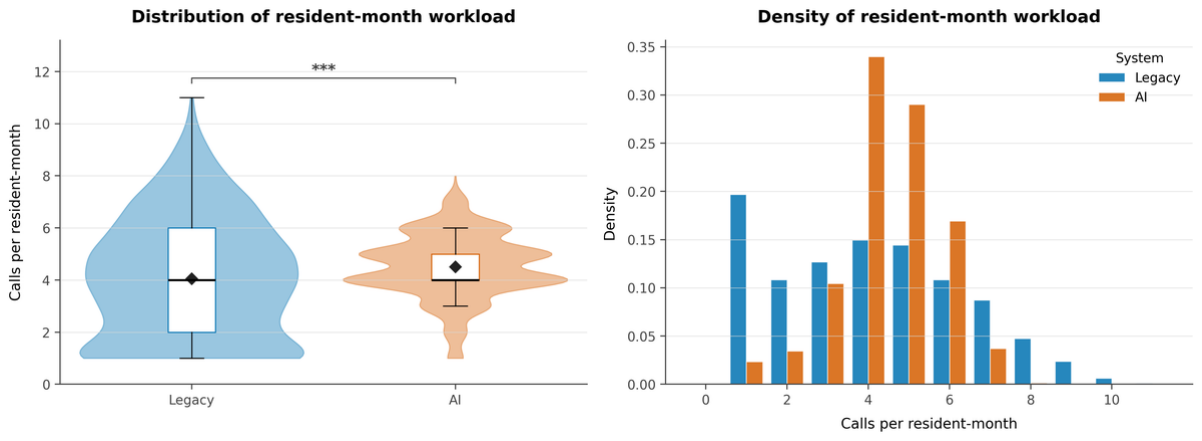
Distribution of total night-call shifts per resident-month, legacy versus AI-based scheduler. The overlaid violins show the empirical distribution of monthly calls per resident. The 2 eras had similar total clinical demand but different per-physician distribution; mean calls per resident-month were modestly higher in the AI era but markedly different dispersion, corresponding to substantially more compact and equitable allocation under the AI-based scheduler.

Across 24 consecutive months, we analyzed 6519 night-call shifts comprising 3246 shifts under the legacy scheduler (107 distinct on-call physicians) and 3273 shifts under the AI-based scheduler (87 distinct on-call physicians), distributed across 803 and 727 resident-months, respectively. Sixty-four physicians appeared in both eras and were tracked across the two systems via a Hebrew-Latin name lookup. The proportion of weekend-tagged shifts was near-identical across eras (legacy 934/3246, 28.8%; AI 935/3273, 28.6%; Pearson *χ*²=0.03, df=1m *P*=.88), and the service mix across the nine night-call positions was not significantly different (omnibus *χ*²_8_=0.78; *P*=.99; Table S4 in Multimedia Appendix 1). Baseline characteristics, including weekend share, are summarized in Table 1, and the per-resident-month call distribution is shown in Figure 1.

**Table 1.** Cohort baseline characteristics for the legacy and AI-based scheduling; 12 months pre- and 12 months postimplementation; January 2024-December 2025.

Variable	Legacy	AI	*P* value
Distinct on-call physicians, n	107	87	—^a^
Total night-call shifts, n	3246	3273	.99^b^
Total weekend-call shifts, n	934	935	.88^b,c^
Calls per resident-month, mean (SD)	4.04 (2.31)	4.50 (1.24)	<.001^d^
Weekend-call share of total shifts (%)	28.8	28.6	.88^b,c^
Weekend calls per resident-month, mean (SD)	1.16 (1.05)	1.29 (0.74)	.008^d^
Total resident-months, n	803	727	—
Cross-era resident matches, n	—	64	—
Distinct night-call services, n	9	9	>.99^b^

a Not applicable.

b *P* value calculated on the basis of chi-square tests.

c *P* value calculated on the basis of Yates tests.

dd *P* value calculated on the basis of Welch *t* tests.

### Workload Distribution and Threshold Exceedances

**Figure 2.**
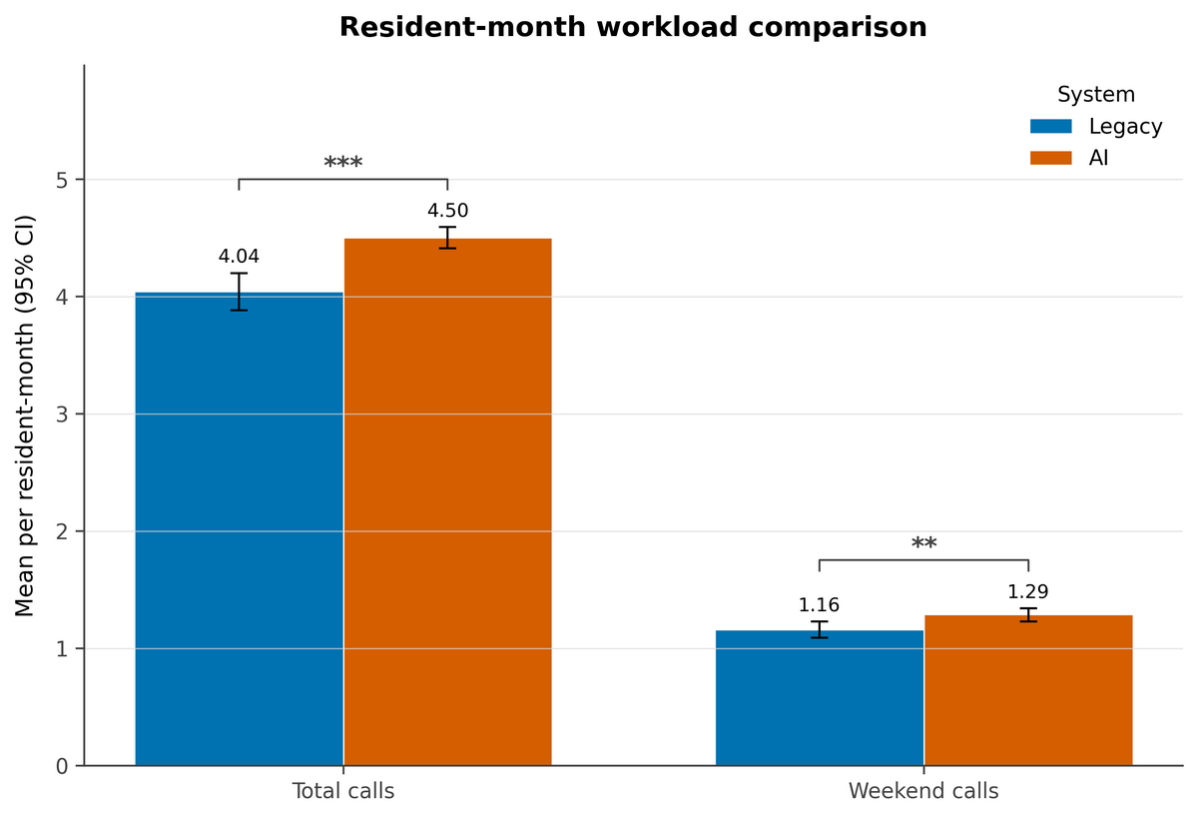
Per-resident-month workload under the legacy and AI-based schedulers. Bars show mean total calls per resident-month and mean weekend calls per resident-month with bootstrap 95% CIs (5000 resamples) for the legacy scheduler (January-December 2024; n=803 resident-months; deep blue) and the AI-based scheduler (January-December 2025; n=727 resident-months; vermillion). *P* values are from 2-sided Welch *t* tests. Mean burden was increased in the AI-era (*Δ*+0.46 calls/resident-month, *P*<.001; *Δ*+0.12 weekend calls per resident-month, *P*=.008).

**Figure 3.**
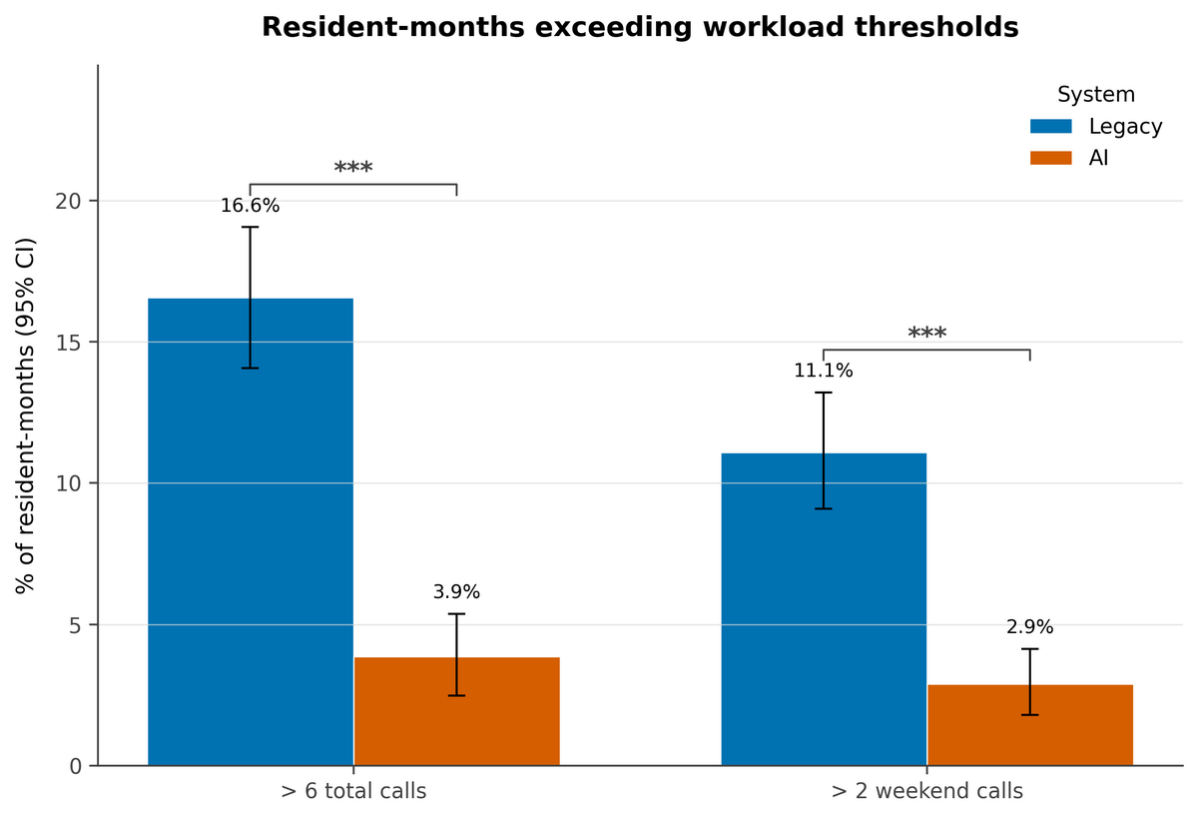
Percentage of resident-months exceeding 2 program-defined workload thresholds (>6 total calls per month; >2 weekend calls per month) under the legacy (n=803 resident-months) and AI-based (n=727 resident-months) schedulers, January 2024-December 2025. Error bars are bootstrap 95% CIs (5000 resamples); ***P<.001 (Pearson chi-square).

Mean calls per resident-month did not decline with the AI-based scheduler (legacy: 4.04, SD 2.31; AI: 4.50, SD 1.24; *Δ*+0.46, 95% CI 0.28‐0.65; Welch *t*_1251_=−4.92 [all Welch *t* values are 2-tailed]; Cohen *d*=−0.25; *P*<.001), and a similarly small mean increase was seen for weekend calls (legacy: 1.16, SD 1.05; AI: 1.29, SD 0.74; *Δ*+0.12; Welch *t*_1445.8_=−2.66; Cohen *d*=−0.13; *P*=.008). Critically, however, the within-period SD was reduced by approximately one-half for both metrics (calls/resident-months: 2.31 → 1.24; weekend calls/resident-months: 1.05 → 0.74), reflecting that the AI-based scheduler distributed an essentially unchanged total monthly clinical demand far more equitably across the on-call physician pool (Figure 2). The proportion of resident-months exceeding the program-defined thresholds fell sharply: 133/803 (16.6%) versus 28/727 (3.9%) for >6 total calls per month (RR 0.24, 95% CI 0.15‐0.34; chi-square *P*<.001; Fisher exact *P*<.001) and 89/803 (11.1%) versus 21/727 (2.9%) for >2 weekend calls per month (RR 0.27, 95% CI 0.16‐0.40; chi-square *P*<.001; Fisher exact *P*<.001). Point estimates and CIs confirm large reductions in overload events (Figure 3; Multimedia Appendix 1).

### Undesirable Shift Sequences

**Figure 4.**
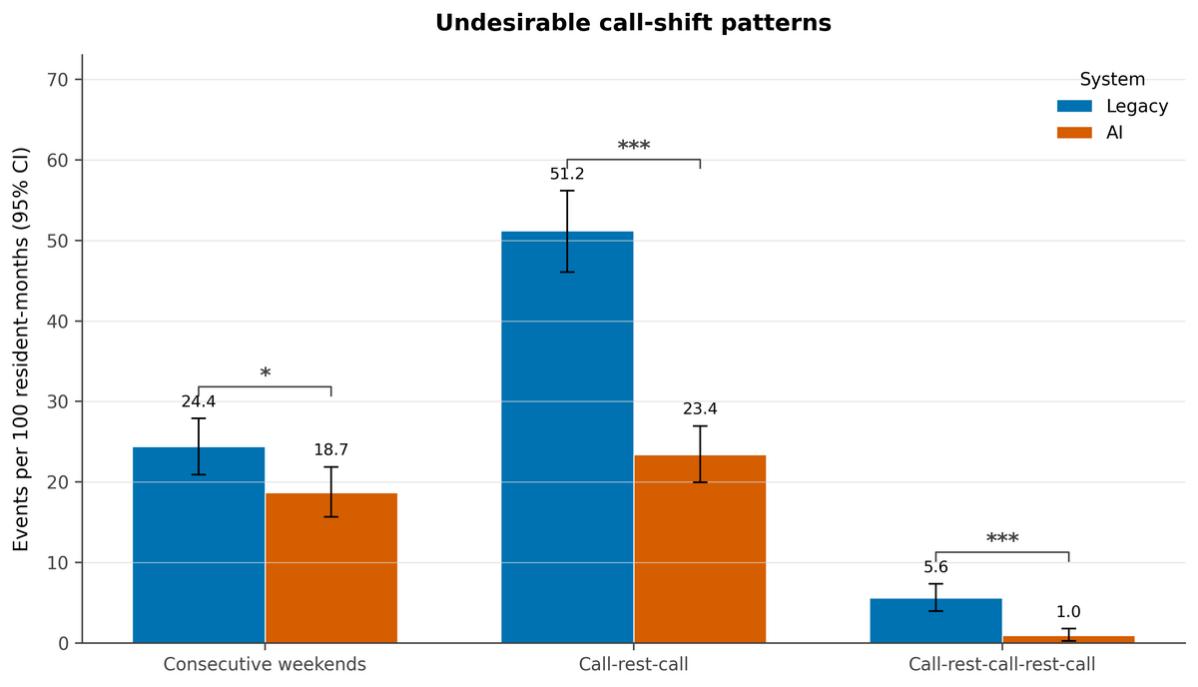
Rates of undesirable on-call shift sequences per 100 resident-months under the legacy (deep blue; n=803 resident-months) and AI-based (vermillion; n=727 resident-months) schedulers, January 2024-December 2025. Error bars are bootstrap 95% CIs (5000 resamples); asterisks denote exact-conditional Poisson rate-ratio tests (*P<.05; ***P<.001).

Rates of undesirable shift sequences declined under the AI-based scheduler (Figure 4). Consecutive weekend on-call episodes, a pattern in which a resident covering a weekend call is again on call the following weekend, fell from 24.4 to 18.7 events per 100 resident-months (RR 0.77, 95% CI 0.62‐0.96; exact-conditional Poisson *P*=.02). Call-rest-call patterns, defined as a call shift followed by exactly one off day before the next call shift, fell from 51.2 to 23.4 events per 100 resident-months (RR 0.46, 95% CI 0.38‐0.55; *P*<.001), and call-rest-call-rest-call (the same pattern extended to a third call shift) fell from 5.6 to 1.0 events per 100 resident-months (RR 0.18, 95% CI 0.06‐0.35; *P*<.001). Together these reductions correspond to approximately one-half fewer double-call episodes and approximately five-fold fewer triple-call episodes per 100 resident-months, patterns that pediatric program leadership had explicitly targeted as fatigue-relevant outcomes in the constraint inventory programmed into the AI-based scheduler (Section 1 in Multimedia Appendix 1).

### Schedule Equity

**Figure 5.**
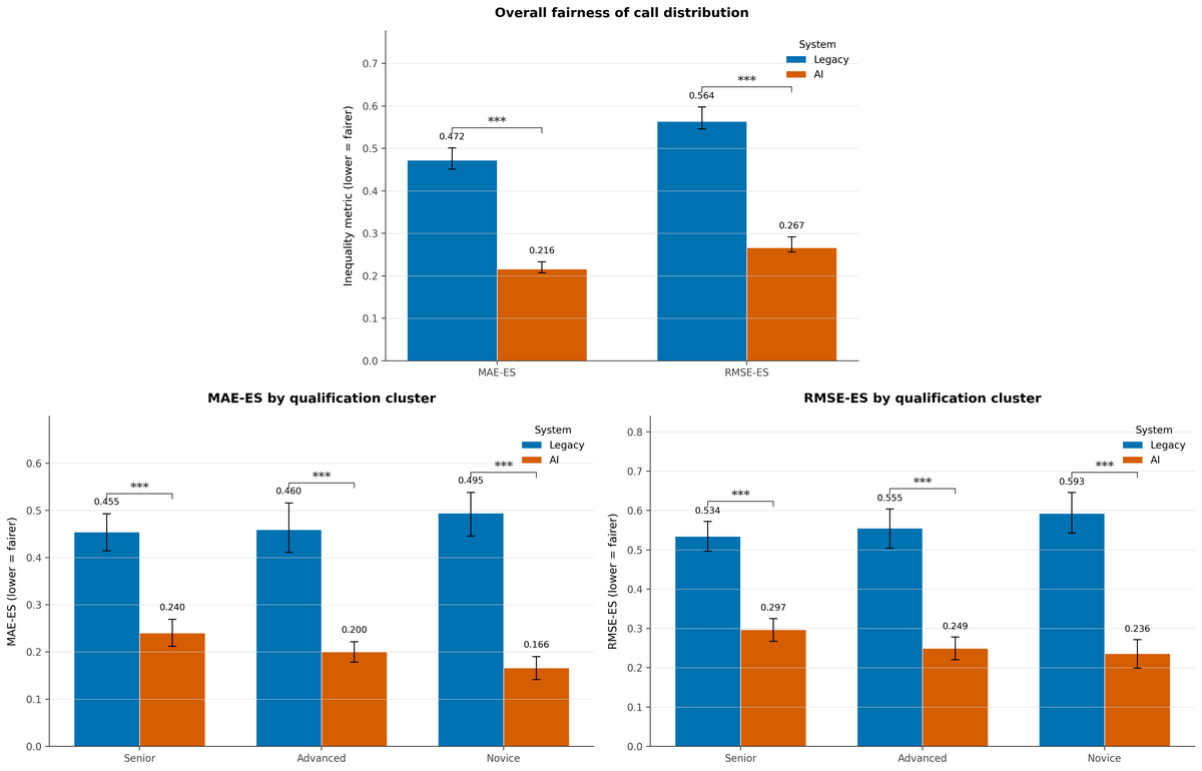
Schedule equity under the legacy and AI-based schedulers. (A) Overall MAE-ES (mean absolute error from equal share) and RMSE-ES (root mean square error from equal share) across all 1530 resident-months (legacy n=803 [deep blue], AI n=727 [vermillion]); lower values indicate more equitable distribution. *P* values are from 2-sided stratified label-permutation tests (25,000 iterations within month-of-year × qualification strata), with bootstrap 95% CIs (5000 stratified resamples). (B) MAE-ES stratified by qualification cluster: Senior (PICU [pediatric intensive care unit] or PCICU [pediatric cardiac intensive care unit] or ED [Senior Emergency Department] 1), Advanced (NICU 1 [neonatal intensive care unit positions 1] or NICU 2 [neonatal intensive care unit positions 2] or ED 2 [junior emergency department call]), and Novice (Ped A [pediatric department A] or Ped B [pediatric department B] or Hem [hematology] and Onc [oncology]). (C) RMSE-ES stratified by qualification cluster. Holm correction was applied across the three clusters. Cluster sizes (legacy → AI): Senior 375 → 299, Advanced 217 → 237, Novice 211 → 191. Lower bars favor the AI-based scheduler; all cluster-level differences were statistically significant at *P*<.001.

Schedule equity, quantified as MAE-ES and RMSE-ES (with smaller values indicating more equitable distribution), improved substantially under the AI-based scheduler both overall and within every qualification cluster (Figure 5; Table 2). Overall MAE-ES decreased by 0.26 shifts (95% CI 0.23‐0.28; permutation *P*<.001) and RMSE-ES by 0.29 shifts (95% CI 0.26‐0.32; *P*<.001). Cluster-stratified analyses, framed as descriptive heterogeneity given that qualification cluster is a posttreatment mediator (see Methods), showed significant improvements in every stratum after Holm correction across the three clusters: Senior (n=375 vs 299) MAE-ES Δ −0.21 (95% CI −0.25 to −0.16; *P*<.001) and RMSE-ES Δ −0.24 (*P*<.001); Advanced (n=217 vs 237) MAE-ES Δ −0.28 (*P*<.001) and RMSE-ES Δ −0.31 (*P*<.001); and Novice (n=211 vs 191) MAE-ES Δ −0.33 (*P*<.001) and RMSE-ES Δ −0.36 (*P*<.001). The fairness gain was therefore robust across qualification levels and was not concentrated in a single cluster. Four preregistered exploratory robustness analyses (Section 4 in Multimedia Appendix 1), spanning four orthogonal axes of distributional behavior, the Lorenz curve and Atkinson(ε) inequality-index sweep, per-resident worst-month tail behavior, intercall temporal burstiness (Goh-Barabási B), and per-physician service-mix variety (Shannon entropy) — supported the same direction of effect on every axis except the service-mix-variety null, which is consistent with the unchanged service-mix proportions between eras (Table S4 in Multimedia Appendix 1).

**Table 2.** Qualification cluster mix in the legacy and AI-based scheduling eras, tertiary pediatric residency, January 2024-December 2025. Clusters are descriptive posttreatment mediators (see methods); cluster mix shifts with both cohort composition and scheduler exposure. Counts and percentages within each era; omnibus and per-cluster z-tests with Holm correction across the three clusters.

Qualification	Legacy, n/N (%)	AI-based, n/N (%)	*P* value (pairwise; Holm-adjusted)
Senior	375/803 (46.7)	299/727 (41.1)	.06
Advanced	217/803 (27.0)	237/727 (32.6)	.05
Novice	211/803 (26.3)	191/727 (26.3)	.99

### Schedule Publication Lead Time

**Figure 6.**
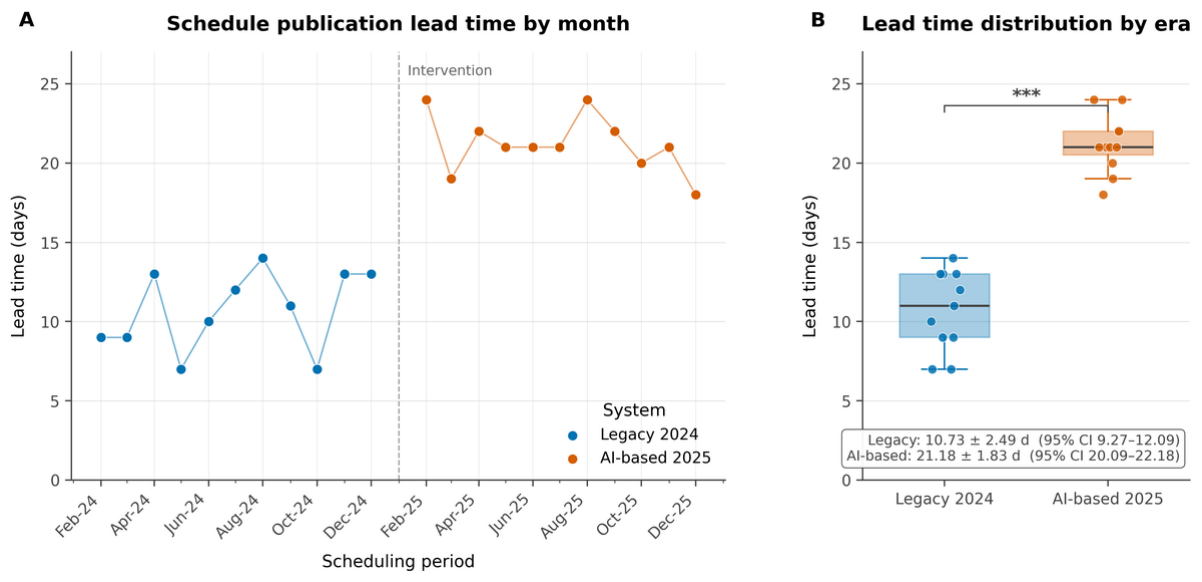
Schedule publication lead time under the legacy and AI-based schedulers, defined as the number of days from schedule publication to the start of the scheduling period it covers (legacy: February-December 2024; AI-based: February-December 2025; n=11 monthly cycles per era). Panel A: lead time by scheduling period; the vertical dashed line marks implementation of the AI-based scheduler in January 2025. Panel B: distribution by era; boxes show the median and IQR, whiskers extend to the most extreme value within 1.5×IQR of the quartiles, and all monthly cycles are overlaid as individual points; means (SD) with bootstrap 95% CIs are inset. ***P<.001 (Welch *t* test).

Publication lead time, the number of days between schedule release and the first day of the upcoming scheduling period, captures the administrative burden of producing a monthly roster. Across 22 monthly cycles (11 legacy and 11 AI-based; February 2024 through December 2024 vs February 2025 through December 2025), the AI-based scheduler approximately doubled lead time relative to the legacy scheduler (Figure 6). Mean lead time increased from 10.7 (SD 2.5) days to 21.2 (SD 1.8) days (*Δ*+10.5 days, 95% CI 8.8‐12.2; 2-tailed Welch *t*_18.37_=11.20; Cohen *d*=4.78; *P*<.001), and the 2 distributions did not overlap (legacy maximum 14 days; AI minimum 18 days; Cliff *δ*=1.00; common-language effect size 100%). The increased lead time was achieved despite no change in upstream demand-planning workflow, consistent with the AI-based scheduler reducing the chief-resident effort required to converge on a publishable roster.

### Interrupted Time-Series Analysis

**Figure 7.**
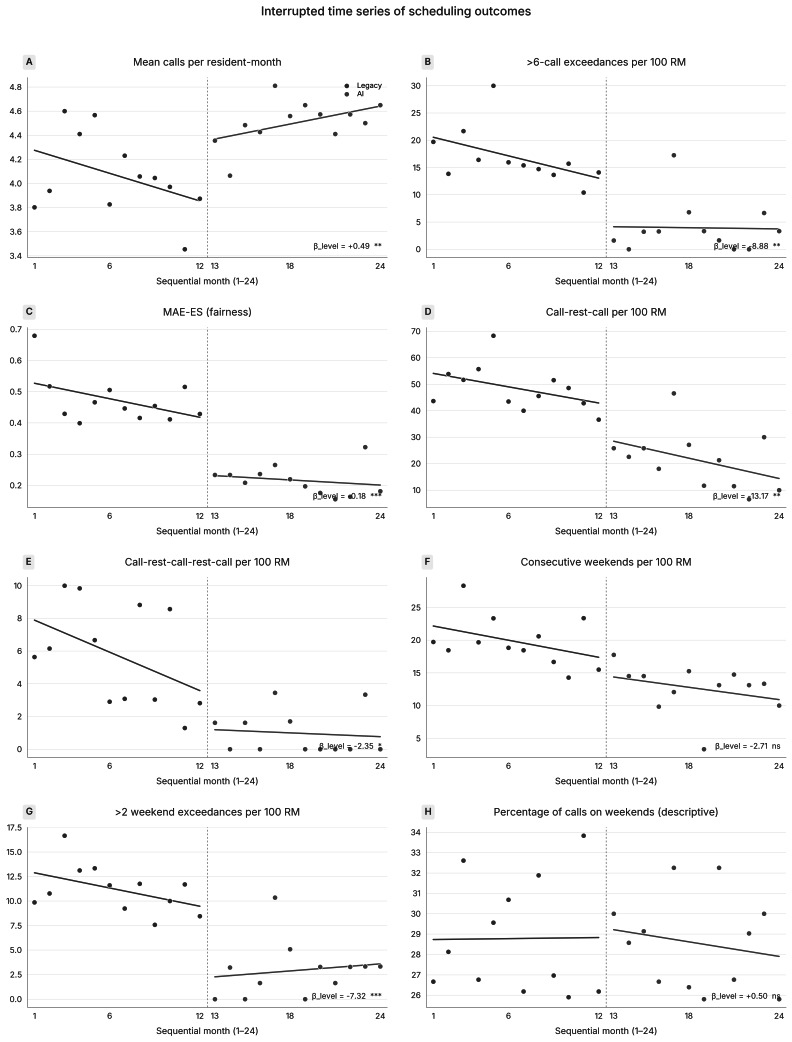
Interrupted time-series analysis of monthly scheduling outcomes, January 2024-December 2025 (24 consecutive months with no inter-system gap). Points are monthly observations (deep blue: legacy scheduler, January–December 2024; vermillion: AI-based scheduler, January–December 2025), red lines the segmented-regression fit, and the vertical dashed line marks implementation between months 12 and 13. Inference uses Newey-West heteroscedasticity- and autocorrelation-consistent standard errors at lag 2; lag-1 and lag-3 sensitivity analyses appear in, Section 2 in Multimedia Appendix 1. Panels: (A) mean total calls per resident-month — β level *+*0.49 (*P*=.004)*;* (B) *>*6-call exceedances per 100 RM *— β* level −8.88 (*P*=.004); (C) schedule equity mean absolute error-estimation standard (MAE-ES) — β level *−*0.18 (*P*<.001)*;* (D) call-rest-call patterns per 100 RM *— β* level −13.17 (*P*=.002); (E) call-rest-call-rest-call patterns per 100 RM — β level *−*2.35 (*P*=.03); (F) consecutive weekend rate per 100 RM *— β* level −2.71 (*P*=.12); (G) >2 weekend-call exceedances per 100 RM — *β* level *−*7.32 *(P<.*001*);* (H) calls falling on weekends (%) — *β *level +0.50 (P=.57). MAE-ES: mean absolute error from equal share; RM: resident-month.

To control for preimplementation secular trends and quantify the immediate level change at intervention, we performed segmented regression on the full 24-month monthly series (Figure 7; Table S1 in Multimedia Appendix 1). Equity (MAE-ES) showed a large, statistically significant improvement at implementation (β_level=−0.18, 95% CI −0.25 to −0.12; *P*<.001) with a stable postimplementation slope (β_slope=+0.007, *P*=.33), indicating a durable level shift rather than a transient effect. Threshold exceedances and high-risk patterns showed similar durable level shifts: >6-call exceedances per 100 resident-months (β_level=−8.88; *P*=.004), call-rest-call patterns (β_level=−13.17 per 100 resident-months; *P*=.002; average postperiod effect −14.88, *P*=.02), call-rest-call-rest-call (β_level=−2.35; *P*=.03), and >2 weekend exceedances (β_level=−7.32 per 100 resident-mo; *P*<.001; average postperiod effect −4.52, *P*=.02). Mean calls per resident-month, in contrast, increased modestly at implementation in the ITS model (β_level=+0.49; *P*=.004). This reflects the smaller postimplementation pool of on-call physicians (87 vs 107) and resident-months (727 vs 803) combined with essentially unchanged total clinical demand (3273 vs 3246 night-call shifts; +0.8%): a smaller denominator with an unchanged numerator mechanically raises the per-resident-month mean. Once all on-call physicians are retained, the AI-based scheduler does not reduce the average per-physician call count but rather compresses its dispersion across physicians (see Workload section). Three quantities did not reach statistical significance after the dataset extension: the level change in the consecutive-weekend rate (β_level=−2.71; *P*=.12), the average postperiod effect for call-rest-call-rest-call patterns (−0.06; *P*=.98), and the level change in the descriptive percentage of weekend calls (β_level+0.50; *P*=.57), consistent with the modest power of a 24-month monthly series with month-to-month noise. Because the AI-effect signature is largely an immediate level shift rather than a postimplementation trend, we report the level coefficient β_level as the primary ITS estimand throughout. Sensitivity analyses at Newey-West heteroscedasticity- and autocorrelation-consistent lags 1 and 3 (Section 2, and Table S2 in Multimedia Appendix 1) yielded substantively identical inference.

### Resident-Reported Experience

**Figure 8.**
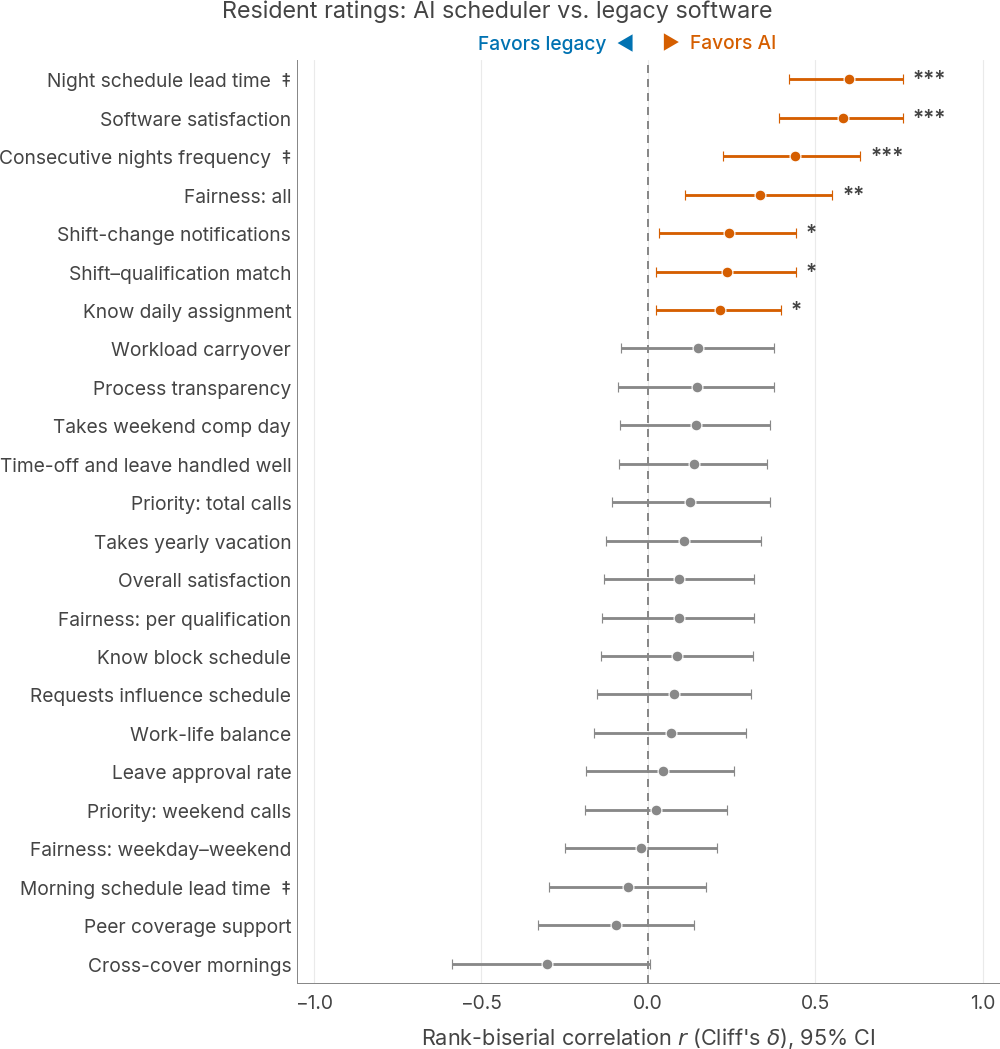
Standardized resident-reported experience with the AI-based versus legacy scheduler. Forest plot. Markers are the rank-biserial correlation r (Cliff's δ) from 2-sided Mann–Whitney U tests comparing AI-era (n=38) with legacy-era (n=47) responses; whiskers are 95% bootstrap CIs (5000 resamples); the dashed line marks no difference. Colored markers are significant at *P*<.05 (vermillion, favors AI; blue, favors legacy); grey markers are nonsignificant. *P<.05; **P<.01; ***P<.001. ‡ Reverse-scored so that higher values indicate a better experience.

Among 47 legacy-era respondents (response rate 47/75, 63%) and 38 AI-era respondents (response rate 38/80, 48%), self-reported experience improved across several scheduling-relevant domains (Figure 8; Section 3 in Multimedia Appendix 1). Software satisfaction increased from 6.77 (SD 2.08) to 8.71 (SD 1.11) on a 10-point scale (Mann-Whitney *U* test; *P*<.001); after reverse scoring, perceived night-call schedule timeliness improved from 3.28 (SD 1.31) to 4.61 (SD 0.55) on a 5-point scale (*P*<.001), and the perceived frequency of consecutive night calls improved from 3.15 (SD 1.43) to 4.24 (SD 1.02) on a 5-point scale (*P*<.001). Perceived equity of workload distribution across all residents increased from 2.98 (SD 1.09) to 3.61 (SD 0.92) (*P*=.006), and residents more often reported knowing where they were scheduled to work (4.38, SD 0.99 → 4.79, SD 0.47, *P*=.03). Several other domains — overall shift satisfaction, process transparency, time-off and leave handling, vacation utilization, workload carry-over, work-life balance, and fairness among similarly qualified residents — showed small AI-legacy mean differences that were not statistically significant (all *P*>.05). Cross-coverage support and peer-coverage availability trended slightly lower under the AI-based scheduler, which we interpret cautiously given the small sample sizes and the seasonal mismatch between the two survey windows (December 2024 and August 2025). A multiple-choice question on preferred shift-trade mechanisms did not differ between eras (*χ*²≈3.7, *P*=.30).

## Discussion

### Principal Findings

Across 24 consecutive months of final executed rosters and 6519 night-call shifts in a tertiary pediatric residency, implementation of an AI-based constraint-programming scheduler was associated with three principal findings. First, schedule equity improved substantially: MAE-ES decreased by 0.26 shifts and RMSE-ES by 0.29 shifts overall, with statistically significant fairness gains in every qualification cluster after Holm correction. Second, high-risk shift sequences and threshold exceedances declined sharply: >6-call months fell from 16.6% (133/803) to 3.9% (28/727), >2 weekend-call months from 11.1% (89/803) to 2.9% (21/727), call-rest-call patterns from 51.2 to 23.4 per 100 resident-months, and call-rest-call-rest-call patterns from 5.6 to 1.0 per 100 resident-months. Third, schedule publication lead time doubled, from a mean of 10.7 (SD 2.5) days to 21.2 (SD 1.8) days, with no overlap between the two distributions. Importantly, mean per-physician call burden did not decrease and in fact rose. Together these findings suggest the operational signal of the AI-based scheduler is to distribute the same overall workload more equitably, with fewer high-risk sequences and possibly less administrative effort for schedulers.

### Comparison to Prior Work

Health care scheduling has historically lagged behind aviation, manufacturing, and logistics in adopting optimization-based decision support [23-26]. Within health care, prior published evaluations have focused largely on patient appointment and operating-room scheduling or on nursing operations [27-30], with comparatively few rigorous quantitative evaluations of physician-residency scheduling in real-world conditions [5-7,11,15,31]. The few residency-focused studies have reported satisfaction improvements but generally relied on subjective endpoints, smaller samples, or shorter horizons. Our 24-month evaluation extends this literature in three directions: (1) by showing the operational benefit of constraint-programming scheduling around distributional equity and reduction in undesirable shift patterns [4,11]; (2) by quantifying the administrative-burden reduction directly through publication lead time, which to our knowledge has not previously been reported with this magnitude (more than 2-fold) in a physician-residency context [32,33]; and (3) by demonstrating uniform fairness gains across employee qualification strata. This is particularly important for highly qualified medical personnel whose schedule is most highly constrained by service-coverage requirements. The directional consistency of effects across our preregistered exploratory robustness analyses (Section 4 in Multimedia Appendix 1) — the Lorenz curve and Atkinson(ε) inequality sweep, per-resident worst-month tail compression, and reduced intercall burstiness — corroborates the central finding.

### Mechanistic Interpretation

Three mechanisms are plausible to explain equitable redistribution of a comparable clinical demand. First, the constraint solver represents the entire monthly assignment problem declaratively and searches for assignments that minimize a composite fairness-aware objective; in contrast, the legacy rule-based scheduler fills shifts sequentially and cannot reverse early local decisions to redress later imbalance. Second, the AI-based platform’s explicit constraint inventory (Section 1 in Multimedia Appendix 1) directly penalizes undesirable call shift patterns defined by schedulers, such as call-rest-call and consecutive-weekend patterns and limits monthly calls per resident, surfaces these preferences to the schedulers through dynamic-statistics dashboards, and lets the reviewer simulate the downstream consequences of any proposed manual edit before publication. Third, by reducing the scheduler effort required to produce a publishable roster, the AI-based platform freed administrative attention for upstream demand planning and qualification management, itself a likely contributor to the more equitable downstream allocation. We interpret the fairness gains, therefore, as the joint product of (1) constraint-aware optimization in the solver and (2) better-informed human-in-the-loop review by chief residents. Distinguishing the marginal contribution of each component requires a controlled comparison and is a priority for future work.

### Generalizability

Our study setting in a single, large tertiary pediatric residency in a national tertiary referral center limits direct generalization. The constraint inventory programmed into the AI-based scheduler is institution-specific (Section 1 in Multimedia Appendix 1), and the magnitude of fairness and lead-time gains may differ in residency programs with smaller pools of on-call physicians, fewer service categories, less complex qualification structures, or different regulatory environments. Several features of our setting do, however, support the broader relevance of these findings: the program covers 9 distinct night-call services with a complex qualification ladder, the on-call physician pool is similar in size to many tertiary residency programs, and the underlying constraint-programming and constraint-based local-search metaheuristic algorithms are domain-general and are not pediatric- or residency-specific. Multisite replication, ideally with a stepped-wedge or cluster-randomized design, would strengthen external validity.

### Limitations

Several limitations of our study warrant explicit acknowledgment, particularly given the well-documented impact of physician fatigue and burnout on patient safety and the resulting need for cautious interpretation of operational improvements that depend on chief-resident effort [34-36]. First, the design is observational and pre- versus postimplementation rather than a randomized prospective trial, so residual confounding from secular trends, cohort composition, regulatory changes, or concurrent quality-improvement activity cannot be excluded. The two study periods were consecutive 12-month windows with comparable service mix, which reduces but does not eliminate the possibility of temporal confounding. The ITS analysis further addressed secular trends, although a monthly series with 12 observations per phase remains modestly powered. Second, the study evaluates final executed rosters that incorporate postsolver and postpublication chief-resident review by schedulers. We cannot isolate the marginal contribution of the constraint solver from the contribution of the platform’s decision-support tools (dynamic statistics, simulation, and AI-prioritized substitutions) or from chief-resident attention; an attention or Hawthorne-like effect during the early postimplementation period is a possibility that we cannot rule out [37]. Third, qualification clusters were inferred from observed shift performance and are therefore posttreatment mediators rather than pretreatment covariates; cluster-stratified equity findings should be read as descriptive heterogeneity rather than as covariate-adjusted causal estimates. Fourth, the AI-based scheduler is a proprietary commercial product, and although we have published its constraint inventory (Section 1 in Multimedia Appendix 1), the underlying solver source code is not publicly available. Fifth, resident surveys captured 47 (63%) and 38 (48%) of approximately 75 and 80 invited residents, respectively; nonresponse bias and a seasonal mismatch between the December 2024 and August 2025 survey windows may affect the precision of cross-era survey comparisons. Sixth, this is a single-center study at a tertiary pediatric facility, and our findings should be replicated in other residency programs and clinical contexts before broader generalization.

### Future Directions

Several directions follow naturally from this work. Multisite, multiprogram, prospective, randomized evaluations are needed to establish external validity and to support causal claims. Longer postimplementation horizons would permit more powerful inference and detection of any posthoneymoon attenuation. Coupling scheduler outputs to downstream measures of trainee well-being, fatigue, and educational attainment—and ultimately to patient-safety outcomes—would translate operational gains into the clinical endpoints that matter most. Finally, beyond classical constraint programming and metaheuristics, methodological frontiers in health care scheduling include reinforcement learning of scheduling policies under realistic clinical simulators [38], generative approaches that produce diverse feasible schedules rapidly, and hybrid systems that use machine-learned models to focus solver search on promising regions of the assignment space. Whether large language models and modern neural networks will prove robust enough for production-grade scheduling decisions remains an open question, but they are already useful for soft-preference elicitation, selective conditional rule-relaxation, and rapid replanning during operational disruptions.

### Conclusions

In a tertiary pediatric residency, implementation of an AI-based constraint-programming scheduler was associated with substantially more equitable distribution of on-call workload across all qualification strata, large reductions in high-risk shift sequences and threshold exceedances, and a doubling of schedule publication lead time, despite no reduction in mean per-physician call burden once all on-call physicians were retained. These findings position AI-driven constraint optimization as a feasible intervention for advancing schedule fairness and reducing administrative burden in graduate medical education. Programs considering similar implementations should focus on rigorous constraint elicitation aligned with program policy, transparent governance of fairness rules, and ongoing auditing of distributional outcomes. Multisite, prospective replication is warranted before generalizing these findings beyond a single tertiary pediatric program.

## Supplementary material

10.2196/88340 Multimedia Appendix 1 Supplementary methods, sensitivity analyses, and exploratory robustness checks.

## Abbreviations

CRC: call-rest-call
ED: emergency department
HAC: heteroscedasticity- and autocorrelation-consistent
ITS: interrupted time-series
MAE-ES: mean absolute error from equal share
ML: machine learning
NICU: neonatal intensive care unit
NP: nondeterministic polynomial time
PCICU: pediatric cardiac intensive care unit
Ped A: pediatric department A
Ped B: pediatric department B
PICU: pediatric intensive care unit
RM: resident-month
RMSE-ES: root mean square error from equal share
RR: risk ratio

## References

[1] Hinostroza Fuentes VG, Karim HA, Tan MJT, AlDahoul N (2025). AI with agency: a vision for adaptive, efficient, and ethical healthcare. Front Digit Health.

[2] Li YH, Li YL, Wei MY, Li GY (2024). Innovation and challenges of artificial intelligence technology in personalized healthcare. Sci Rep.

[3] Lip G, Novak A, Goyen M, Boylan K, Kumar A (2024). Adoption, orchestration, and deployment of artificial intelligence within the National Health Service—facilitators and barriers: an expert roundtable discussion. BJR Artificial Intell.

[4] Said AB, Mouhoub M (2024). Machine learning and constraint programming for efficient healthcare scheduling. arXiv.

[5] Porche K, Mohan A, Dow J (2024). Automated and optimized neurosurgery scheduling system improves resident satisfaction. Neurosurgery.

[6] Howard FM, Gao CA, Sankey C (2020). Implementation of an automated scheduling tool improves schedule quality and resident satisfaction. PLoS One.

[7] Chu E, Hindle AK, Abeledo H (2021). An equitable electronic scheduling system for anesthesiology residents: a quality improvement project. J Educ Perioper Med.

[8] Perelstein E, Rose A, Hong YC, Cohn A, Long MT (2016). Automation improves schedule quality and increases scheduling efficiency for residents. J Grad Med Educ.

[9] Smalley HK, Keskinocak P (2016). Automated medical resident rotation and shift scheduling to ensure quality resident education and patient care. Health Care Manag Sci.

[10] Lu Y (2025). A multimodal deep reinforcement learning approach for IoT-driven adaptive scheduling and robustness optimization in global logistics networks. Sci Rep.

[11] Cohn A, Root S, Kymissis C, Esses J, Westmoreland N (2009). Scheduling medical residents at Boston University School of Medicine. Interfaces (Providence).

[12] Bard JF, Shu Z, Leykum L (2013). Monthly clinic assignments for internal medicine housestaff. IIE Trans Healthc Syst Eng.

[13] Brown N, Pilarski A (2014). EM residency schedule improvement with introduction of a computer-assisted system. West J Emerg Med.

[14] Hollenbach SJ, Harrington A, Duecy E (2018). Automated residency scheduling: impacts on resident satisfaction and wellness [6C]. Obstet Gynecol.

[15] Oppenheim BE (1989). Computer-assisted radiology resident rotation scheduling. Invest Radiol.

[16] Nwanaji-Enwerem JC, Ehrhardt TF, Gordon B (2024). Considering burnout and well-being: emergency medicine resident shift scheduling platform and satisfaction insights from a quality improvement project. Health Care (Don Mills).

[17] Knight DRT, Aakre CA, Anstine CV (2023). Artificial intelligence for patient scheduling in the real-world health care setting: a metanarrative review. Health Policy Technol.

[18] Vedaa Ø, Mørland E, Larsen M (2017). Sleep detriments associated with quick returns in rotating shift work: a diary study. J Occup Environ Med.

[19] Sumrall WD, Oury JV, Gilly GM (2025). Enhancing physician satisfaction and patient safety through an artificial intelligence-driven scheduling system in anesthesiology. Ochsner J.

[20] Gilad D, Farbstein-Aljanati T, Afek A, Pessach IM, Ashkenazi M (2025). AI-driven shift scheduling: insights from a pilot in Safra Children’s Hospital. Isr Med Assoc J.

[21] Bernal JL, Cummins S, Gasparrini A (2017). Interrupted time series regression for the evaluation of public health interventions: a tutorial. Int J Epidemiol.

[22] Newey WK, West KD (1987). A simple, positive semi-definite, heteroskedasticity and autocorrelation consistent covariance matrix. Econometrica.

[23] Xu Y, Wandelt S, Sun X (2024). Airline scheduling optimization: literature review and a discussion of modelling methodologies. Intell transp infrastruct.

[24] Del Gallo M, Mazzuto G, Ciarapica FE, Bevilacqua M (2023). Artificial intelligence to solve production scheduling problems in real industrial settings: systematic literature review. Electronics (Basel).

[25] Ding J, Chen M, Wang T, Zhou J, Fu X, Li K (2023). A survey of AI-enabled dynamic manufacturing scheduling: from directed heuristics to autonomous learning. ACM Comput Surv.

[26] Abdalkareem ZA, Amir A, Al-Betar MA, Ekhan P, Hammouri AI (2021). Healthcare scheduling in optimization context: a review. Health Technol.

[27] Kang HW, Kim J, Kim KJ (2025). Shift nurses’ work quality and job satisfaction after implementing the Inha University hospital nursing AI scheduling system (IH-NASS). BMC Nurs.

[28] Liebowitz SH, Robertson M (2024). Revolutionizing schedules: the power of AI in physician practices. Front Health Serv Manage.

[29] Leung F, Lau YC, Law M, Djeng SK (2022). Artificial intelligence and end user tools to develop a nurse duty roster scheduling system. Int J Nurs Sci.

[30] Wood KV, Frings D, Flood C, Thomas N (2025). Artificial intelligence machine learning-driven outpatient appointment management: a qualitative study on acceptability. Digit Health.

[31] Shahraki N, Sir MY, Prindle T, Ramar K (2022). A decision-support system to schedule rotations for trainees. ATS Sch.

[32] Erhard M, Schoenfelder J, Fügener A, Brunner JO (2018). State of the art in physician scheduling. Eur J Oper Res.

[33] Garg M, Kompala T, Hurley M, López L (2022). Characterization of internal medicine chief resident administrative, educational, and clinical experiences. JAMA Netw Open.

[34] Brunsberg KA, Landrigan CP, Garcia BM (2019). Association of pediatric resident physician depression and burnout with harmful medical errors on inpatient services. Acad Med.

[35] Landrigan CP, Rothschild JM, Cronin JW (2004). Effect of reducing interns’ work hours on serious medical errors in intensive care units. N Engl J Med.

[36] Landrigan CP, Rahman SA, Sullivan JP (2020). Effect on patient safety of a resident physician schedule without 24-hour shifts. N Engl J Med.

[37] McCambridge J, Witton J, Elbourne DR (2014). Systematic review of the Hawthorne effect: new concepts are needed to study research participation effects. J Clin Epidemiol.

[38] Wu Q, Han J, Yan Y, Kuo YH, Shen ZJM (2025). Reinforcement learning for healthcare operations management: methodological framework, recent developments, and future research directions. Health Care Manag Sci.

[39] Optimizing on-call schedule equity and reducing administrative burden in a pediatric residency: a 24-month before-after evaluation of an AI-based constraint-optimization scheduler. Zenodo.

