# Supplementary material for: Evaluation of an AI-Based Constraint-Optimization Scheduler to Optimize On-Call Schedule Equity and Reduce Administrative Burden in a Pediatric Residency: Retrospective Comparative Study
Source: J Med Internet Res. 2026 Jul 31;28:e88340. doi: 10.2196/88340 (PMC13426424; doi:10.2196/88340)
Supplement: Multimedia Appendix 1 [file jmir-v28-e88340-s001.docx]

**Multimedia Appendix 1. Supplementary methods, sensitivity analyses, and exploratory robustness checks**

Manuscript: Optimizing On-Call Schedule Equity and Reducing Administrative Burden in a Pediatric Residency: A 24-Month Before-After Evaluation of an AI-Based Constraint-Optimization Scheduler (Gilad et al., JMIR submission #88340).

**Section 1. Description of the AI-based scheduling system: solver class, constraint inventory, and human-in-the-loop review workflow**

**1.1 Solver class and architecture**

The AI-based scheduler (Equina; https://www.equinascheduling.com/) is a healthcare-adapted constraint-programming platform built on the open-source Timefold solver, which provides a constraint-based local-search (CBLS) metaheuristic implementing variants of late-acceptance hill climbing, simulated annealing, and tabu search. Unlike a rule-based heuristic that fills shifts sequentially under a fixed priority order, a constraint solver represents the entire monthly assignment problem declaratively and searches the space of feasible assignments to minimize a weighted composite objective covering coverage adequacy, distributional fairness, sequence avoidance, and resident-level preferences. Each shift assignment is evaluated against the full constraint inventory simultaneously, and the solver is allowed to revise earlier local choices to satisfy later constraints; a property that rule-based autoschedulers structurally lack.

**1.2 Hard constraints (must be satisfied for a roster to be feasible)**

| ID | Domain | Specification |
| --- | --- | --- |
| H1 | Coverage Demand | Each of the 9 night-call services (PICU, PCICU, NICU 1, NICU 2, ED 1, ED 2, Hem/Onc, Ped A, Ped B) must be staffed by exactly one qualified physician for every calendar date. |
| H2 | Eligibility | Each shift assignment must respect the assigned physician's documented qualification (per-shift authorization plus current per-block-rotation eligibility). |
| H3 | Overlapping shift conflicts | No physician may be assigned to two services on the same calendar date. |
| H4 | Block schedule | Physicians assigned to specific block rotations (e.g., outside-rotation, vacation block) are excluded from on-call eligibility for that block. Note: block scheduling rules were primarily used for morning/evening shifts associated with their respective blocks, whereas call shifts were assigned based on resident qualification. |
| H5 | Time-off, Time-on and leave requests | Approved time-off, leave, and personal-event windows are blocked. Employees were allowed to submit wanted call shift time slots (“Time on”). |
| H6 | Mandatory rest | A 24-hour rest window after each call shift before the next call shift. |
| H7 | Cap on monthly weekday calls | Per contract; default 4-6 weekday calls per month. |
| H8 | Cap on monthly weekend calls | Per contract; default 1-3 weekend calls per month. |
| H9 | Call-rest-call-rest-call avoidance | Penalize the same alternating pattern extended to a third call. |

**1.3 Soft constraints (penalized in the composite objective; weights tuned by chief residents)**

| ID | Domain | Specification |
| --- | --- | --- |
| S1 | Workload equity | Penalize the deviation of each physician's monthly call count from the equal-share expectation, weighted by qualification cluster. with historical consideration during the AI-period. |
| S2 | Weekend equity | Penalize the deviation of each physician's monthly weekend-call count from equal-share expectation. with historical consideration during the AI-period. |
| S3 | Consecutive weekend avoidance | Penalize a physician on call on consecutive weekends within the same scheduling period. |
| S4 | Call-rest-call avoidance | Penalize sequences of call → exactly one off day → call. |
| S5 | Holiday equity | Penalize the deviation of each physician's monthly holiday call shift count from the equal-share expectation, weighted by qualification cluster, with historical consideration during the AI-period. |
| S6 | Friday/Saturday split balance | Maintain comparable Friday vs Saturday assignment counts across qualification clusters. |
| S7 | Service-mix variety | Penalize repeated assignment of the same physician to the same service across consecutive months when alternative qualified physicians are available. |
| S8 | Qualification-preferred assignments | Reward assignment of senior-eligible physicians to senior services (PICU/PCICU/ED 1) and reserve advanced-only services for advanced-eligible physicians. |
| S9 | Personal preferences | Reward honoring resident-submitted preferences for specific dates or services where feasible. |
| S10 | Pair preferences | Soft preference for or against specific co-call pairings. |
| S11 | Historical fairness | Adjust weights using calls performed in prior months (rolling lookback) so that residents under-served historically receive marginal preference. |

**1.4 Qualification mappings**

Qualification eligibility for each of the 9 night-call services is configured per physician and updated as residents accumulate service-specific exposure. The descriptive qualification clusters used in the equity analyses are defined as follows (the most demanding shift performed up to and including the index month determines cluster membership):

| Cluster | Defining shift exposure |
| --- | --- |
| Senior | PICU Call OR PCICU Call OR ED 1 Call |
| Advanced | NICU 1 Call OR NICU 2 Call OR ED 2 Call (without yet meeting Senior criteria) |
| Novice | Ped A Call OR Ped B Call OR Hem/Onc Call only |

**1.5 Human-in-the-loop review workflow**

Both the legacy and AI-based schedulers operate within a structured chief-resident review workflow. The steps below describe the AI-based platform's workflow at the study site:

Step 1 — Demand assembly. The chief residents finalize the monthly demand template (services, dates, eligibility windows) and confirm the active physician pool with updated monthly qualifications as residents progress in training, including any one-off stand-ins.

Step 2— Simulation. Before approving employee submitted requests, chief residents use the platform's schedule-simulation feature to evaluate the downstream consequences of proposed edits (effect on fairness metrics, sequence rates, downstream cluster mix).

Step 3— Solver run. The constraint solver is invoked with the current constraint weights and produces a candidate roster. Solver runtime is typically 30 seconds to 5 minutes, depending on problem size.

Step 4 — Dynamic-statistics review. Chief residents inspect the platform's real-time dashboards covering per-physician call counts, weekend-call counts, qualification-cluster distribution, and high-risk-pattern incidence. Items flagged outside acceptable ranges are queued for review.

Step 5 — Manual edits. Edits are committed and the solver is re-invoked to re-optimize within the user-pinned constraints.

Step 6 — Publication. The final roster is published to the resident-facing web and mobile applications.

Step 7 — Post-publication adjustments. Last-minute illness, personal events, or service demand changes are handled via AI-prioritized substitution recommendations: the platform ranks candidate substitutes by qualification, current monthly load, and historical fairness, and chief residents accept, modify, or override the recommendation.

**1.6 Reproducibility note**

The solver source code is proprietary; this manuscript reports an evaluation of its real-world implementation, not a reproduction of the algorithm itself. The complete constraint inventory above is sufficient to guide replication of the constraint structure on other constraint-programming platforms. The evaluation pipeline (Python source code and analysis scripts) is available from a publicly disclosed Zenodo repository **https://doi.org/10.5281/zenodo.20365209.**

**Section 2. Statistical methods supplement: ITS lag sensitivity, permutation strata, qualification-mediator framing, and human-in-the-loop quantification**

**2.1 ITS specification and Newey-West HAC lag-sensitivity analysis**

The interrupted time-series (ITS) analysis used a single segmented-regression series spanning Time_Month 1-24 (Time_Month 1-12 = Legacy era, January-December 2024; Time_Month 13-24 = AI era, January-December 2025), with the intervention boundary between Time_Month 12 and 13. The segmented-regression model was Outcome = β₀ + β₁·Time + β₂·Intervention + β₃·Post_Time, where Intervention is 0 in the pre-period and 1 in the post-period and Post_Time is 0 in the pre-period and (Time_Month − 12) in the post-period. The primary estimand was β₂ (immediate level change at implementation); the secondary estimand was the average post-period effect β₂ + 6.5·β₃, with standard errors derived from the full coefficient covariance matrix.

**Table S1. Interrupted time-series segmented-regression coefficients for all eight outcomes, January 2024 – December 2025 (24 consecutive monthly observations; Newey-West HAC, lag 2; primary specification).**

| **Panel** | **Outcome** | **β_level (95% CI)** | **P (β_level)** | **Pre-period slope (β₁)** | **Post-period slope β₃ (P)** | **Avg post-period effect (95% CI; P)** | **R²_adj** |
| --- | --- | --- | --- | --- | --- | --- | --- |
| A | Mean calls per resident-month | +0.49 (95% CI +0.16, +0.82) | P=.004 | −0.04 | +0.06 (P=.04) | +0.90 (95% CI +0.31, +1.48; P=.007) | 0.45 |
| B | >6-call exceedances per 100 RM | −8.88 (95% CI −14.84, −2.92) | P=.004 | −0.68 | +0.65 (P=.08) | −4.68 (95% CI −9.92, +0.55; P=.09) | 0.65 |
| C | MAE-ES (fairness) | −0.18 (95% CI −0.25, −0.12) | P<.001 | −0.01 | +0.01 (P=.33) | −0.14 (95% CI −0.28, +0.00; P=.07) | 0.82 |
| D | Call-rest-call per 100 RM | −13.17 (95% CI −21.63, −4.72) | P=.002 | −1.02 | −0.26 (P=.72) | −14.88 (95% CI −26.72, −3.04; P=.02) | 0.69 |
| E | Call-rest-call-rest-call per 100 RM | −2.35 (95% CI −4.49, −0.22) | P=.03 | −0.39 | +0.35 (P=.04) | −0.06 (95% CI −3.87, +3.76; P=.98) | 0.55 |
| F | Consecutive weekends per 100 RM | −2.71 (95% CI −6.14, +0.73) | P=.12 | −0.43 | +0.12 (P=.67) | −1.93 (95% CI −6.40, +2.55; P=.41) | 0.50 |
| G | >2 weekend exceedances per 100 RM | −7.32 (95% CI −10.23, −4.40) | P<.001 | −0.31 | +0.43 (P=.04) | −4.52 (95% CI −7.98, −1.06; P=.02) | 0.71 |
| H | % calls on weekends (descriptive) | +0.50 (95% CI −1.22, +2.23) | P=.57 | +0.01 | −0.13 (P=.38) | −0.33 (95% CI −3.30, +2.65; P=.83) | -0.13 |

**Table S2. Lag-sensitivity of β_level under Newey-West HAC at lags 0–3 (lag 0 corresponds to classical OLS variance; lags 1, 2, and 3 use HAC standard errors; the manuscript's primary specification is lag 2).**

Inference used Newey-West heteroscedasticity- and autocorrelation-consistent (HAC) standard errors. The primary specification used lag = 2; we report inference at lag 1 and lag 3 here for sensitivity. With a 24-month series, lags greater than 3 absorb a substantial fraction of effective degrees of freedom and are not recommended; lag 0 corresponds to the OLS baseline.

| Outcome (panel) | OLS (lag 0) | Lag 1 | Lag 2 (primary) | Lag 3 |
| --- | --- | --- | --- | --- |
| MAE-ES (Panel C) | −0.18 (P=.001) | −0.18 (P<.001) | −0.18 (P<.001) | −0.18 (P<.001) |
| >6-call exceedances/100 RM (B) | −8.88 (P=.037) | −8.88 (P=.003) | −8.88 (P=.004) | −8.88 (P=.002) |
| Call-rest-call/100 RM (D) | −13.17 (P=.101) | −13.17 (P=.008) | −13.17 (P=.002) | −13.17 (P<.001) |
| >2 weekend exceedances/100 RM (G) | −7.32 (P=.003) | −7.32 (P<.001) | −7.32 (P<.001) | −7.32 (P<.001) |
| Mean calls per resident-month (A) | +0.49 (P=.032) | +0.49 (P=.004) | +0.49 (P=.004) | +0.49 (P<.001) |

Conclusion: at the primary (lag 2) specification and at lags 1 and 3, inference is substantively unchanged for every outcome reported in the manuscript. The single exception is Panel D (call-rest-call) at lag 0 (classical OLS): the homoskedasticity-only standard error widens the confidence interval enough that the level effect is not statistically significant (P=.101), whereas the HAC-corrected lag-1, lag-2, and lag-3 P-values are all <.01. This reflects the imprecision of OLS standard errors on a 24-month series with month-to-month variability and is not the intended primary inference. The primary lag-2 specification was retained for the manuscript because it provides a conservative balance between unbiased SE estimation and degrees-of-freedom preservation in a 24-month series.

**2.2 Stratified label-permutation inference for fairness outcomes**

For MAE-ES and RMSE-ES we used two-sided stratified label-permutation tests (25,000 iterations) within month-of-year × qualification-cluster strata. With three clusters (Senior, Advanced, Novice) and 12 months, the cluster-stratified analysis used 36 strata; the overall analysis used 12 month-of-year strata with cluster pooling. Permutations preserve the empirical distribution of monthly call counts under the null hypothesis of era-label exchangeability and avoid the anti-conservative bias that arises if Era labels are shuffled globally across all months (because Legacy and AI live in disjoint time windows). Bootstrap 95% confidence intervals were computed from 5,000 stratified resamples (resampling resident-months with replacement within each stratum). Holm correction was applied across the three clusters per outcome.

**2.3 Qualification cluster as post-treatment mediator: framing rationale**

Qualification cluster is determined by the most demanding service a physician has performed up to and including the index month. Performance of demanding services is itself a function of scheduler exposure: a constraint-aware scheduler will assign senior-eligible residents to senior services more uniformly than a rule-based heuristic, which in turn changes the rate at which junior residents accumulate qualifications. Cluster membership therefore reflects, at every cross-section, both pre-treatment cohort composition and accumulated post-treatment scheduler exposure. We treat cluster as a post-treatment mediator and report cluster-stratified equity results as descriptive heterogeneity rather than as covariate-adjusted causal effects. Cross-era differences in cluster mix (Table 2) reflect this combined influence and are interpreted as joint outcomes, not as confounders to be controlled.

**2.4 Reproducibility**

All randomized steps (permutation tests, bootstrap resampling) used np.random.seed(42). The evaluation pipeline (Python source code and analysis scripts) is available from a publicly disclosed Zenodo repository **https://doi.org/10.5281/zenodo.20365209.**

**2.5 Resident-month-level threshold-exceedance rates**

**Table S3. Resident-month-level threshold-exceedance rates under the legacy and AI-based schedulers, January 2024 – December 2025. Counts are resident-months exceeding each program-defined threshold; percentages are with 95% Wilson confidence intervals; relative risks compare AI to legacy; Pearson χ² uses Yates' continuity correction.**

| **Outcome** | **Legacy n/N (%; 95% CI)** | **AI n/N (%; 95% CI)** | **RR AI/Legacy (95% CI)** | **χ²_Yates (P)** | **Fisher OR (P)** |
| --- | --- | --- | --- | --- | --- |
| >6 total calls per resident-month | 133 / 803 (16.6%; 95% CI 14.1, 19.1) | 28 / 727 (3.9%; 95% CI 2.5, 5.4) | 0.24 (95% CI 0.150, 0.338) | 64.1 (P<.001) | 0.20 (P<.001) |
| >2 weekend calls per resident-month | 89 / 803 (11.1%; 95% CI 9.1, 13.2) | 21 / 727 (2.9%; 95% CI 1.8, 4.1) | 0.27 (95% CI 0.156, 0.400) | 37.2 (P<.001) | 0.24 (P<.001) |

**Section 3. Resident questionnaire instrument and response rates**

**3.1 Instrument development**

The questionnaire was developed by the residency program leadership for this study. Items were drafted to cover the scheduling-relevant experience domains identified by chief residents and program faculty: software satisfaction, perceived schedule fairness (overall and within similarly qualified residents), perceived workload distribution, call-sequence experience, leave and time-off handling, schedule-publication timeliness, work-life balance, and cross-coverage support. The instrument was pilot-tested informally with three chief residents prior to the legacy-era distribution. The instrument was administered in Hebrew; the English translation reproduced below was prepared by the authors. Items use 5-point Likert scales unless otherwise noted; the software-satisfaction item uses a 10-point scale. Negatively framed items (frequency of consecutive nights; schedule lead-time satisfaction) are reverse-scored as indicated.

**3.2 Response rates**

The legacy-era survey was distributed by Google Forms link on December 14-15, 2024 to approximately 75 active residents. 47 responded (response rate 47/75, 63%). The post-implementation survey was distributed on August 22-27, 2025 to approximately 80 active residents; 38 responded (response rate 38/80, 48%). Denominators are approximate because residents joining mid-month or rotating off-service are variably included in the eligible-resident roster at the moment of survey distribution. Both surveys were anonymous; demographic identifiers were not collected, precluding paired analysis between eras and limiting our ability to assess nonresponse bias by postgraduate year.

| Item | Legacy era | AI-based era |
| --- | --- | --- |
| Distribution date | December 14-15, 2024 | August 22-27, 2025 |
| Eligible residents (approximate) | ~75 | ~80 |
| Respondents | 47 | 38 |
| Response rate | 63% | 48% |
| Distribution channel | Google Forms | Google Forms |
| Anonymity | Anonymous | Anonymous |
| Identifying data captured | None | None |

**3.3 Survey instrument (24 items)**

Q1. How content are you overall with your shift schedule? (1-5)

Q2. How content are you with the scheduling software? (1-10)

Q3. How transparent is the scheduling process? (1-5)

Q4. The schedule balances the workload between ALL residents well. (1-5)

Q5. The schedule balances the workload between SIMILARLY QUALIFIED residents well. (1-5)

Q6. The weekend and weekday shift distribution is fair between residents. (1-5)

Q7. The workload I was given in a given month is taken into consideration when planning the next month. (1-5)

Q8. The most important balance for me is the WEEKEND call count between similarly qualified residents. (1-5)

Q9. How often do you work consecutive night calls? (1-5; reverse-scored)

Q10. The current scheduling software effectively considers my time off and leave requests. (1-5)

Q11. What proportion of leave requests you make is approved? (1-5)

Q12. I manage to take my yearly vacation leave days in a given calendar year. (1-5)

Q13. I manage to take my compensatory rest day for weekend calls. (1-5)

Q14. The requests I submit with the scheduling solution significantly impact my published schedule. (1-5)

Q15. My shift schedule allows for reasonable work-life balance. (1-5)

Q16. The morning shift schedule is published later than desired. (1-5; reverse-scored)

Q17. The night on-call shift schedule is published later than desired. (1-5; reverse-scored)

Q18. I always know where I work according to the published schedule in the software. (1-5)

Q19. I know my planned residency block schedule. (1-5)

Q20. For PGY-2 and above: How often do you cross-cover a morning shift outside of your assigned rotation? (1-5)

Q21. I'm assigned to shifts that fit my clinical abilities and qualifications. (1-5)

Q22. When I need replacement, my colleagues help me out. (1-5)

Q23. I'm always updated about shift changes with a direct message from the chief resident. (1-5)

Q24. Preferred mechanism for trading/swapping shifts (multiple choice).

**3.4 Reverse-scoring key**

Items Q9, Q16, and Q17 are negatively framed. Raw responses on the 1-5 scale were reverse-scored using R' = 6 − R so that, for all items, higher values uniformly indicate better experience.

**3.5 Limitations**

We acknowledge two limitations of these survey data. First, the December 2024 (winter respiratory surge) and August 2025 (summer) survey timings differ in seasonal context, which may affect items related to workload perception, fatigue, and call frequency. Second, anonymity precluded paired analysis and prevents stratification by postgraduate year. Both points are addressed in the manuscript Limitations subsection.

**Section 4. Exploratory robustness analyses**

**4.0 Overview**

We performed four exploratory robustness analyses to test whether the manuscript's central distributional-equity finding holds under alternative formulations of inequality, tail behavior, regularity, and service-mix variety. Each analysis was performed on the same January 2024-December 2025 panel used for the primary manuscript analyses. Permutations were stratified within month-of-year × qualification-cluster strata (10,000 iterations); bootstrap 95% confidence intervals used 5,000 stratified resamples; Holm correction was applied across the three clusters within each analysis. We did not apply a global multiplicity correction across the four analyses; we present these results as exploratory robustness checks rather than as confirmatory hypothesis tests.

The four analyses cover four orthogonal axes of distributional behavior: summary inequality across the full call-count distribution; the per-resident worst-month tail; inter-call temporal regularity; and the diversity of services each physician experiences.

**4.1 Lorenz / inequality-index family with Atkinson(ε) sweep**

We computed the Lorenz curve and six summary inequality indices over the resident-month call-count distribution: the Gini coefficient, the Hoover (Robin Hood) index, the Palma ratio (top 10% / bottom 40% share), and the Atkinson family at three inequality-aversion parameters (ε = 0.5, 1.0, 2.0). The Atkinson sweep is informative because increasing ε progressively up-weights inequality at the bottom of the distribution; consistency of the sign and magnitude of the change across ε values indicates that the equity gain is not driven solely by behavior at one tail.

All indices declined substantially under the AI-based scheduler. The Hoover index - the smallest fraction of total calls that would need to be re-distributed to achieve perfect equality, fell from 0.237 to 0.110 (P<.001 under stratified permutation), corresponding to a 54% reduction in the share-redistribution required. The Palma ratio fell from 1.17 to 0.47 (P<.001). The Gini coefficient and all three Atkinson(ε) indices declined with P<.001 under stratified permutation; cluster-level effects mirrored the overall pattern in every cluster after Holm correction.

| Inequality index | Legacy | AI | Δ (AI − Legacy) | P (permutation) |
| --- | --- | --- | --- | --- |
| Hoover index (Robin Hood) | 0.237 | 0.110 | −0.13 | <.001 |
| Palma ratio (top 10% / bot 40%) | 1.17 | 0.47 | −0.70 | <.001 |
| Gini coefficient | ≈ 0.32 | ≈ 0.15 | ≈ −0.18 | <.001 |
| Atkinson(ε=0.5) | ≈ 0.09 | ≈ 0.02 | ≈ −0.07 | <.001 |
| Atkinson(ε=1.0) | ≈ 0.19 | ≈ 0.05 | ≈ −0.14 | <.001 |
| Atkinson(ε=2.0) | ≈ 0.38 | ≈ 0.12 | ≈ −0.26 | <.001 |


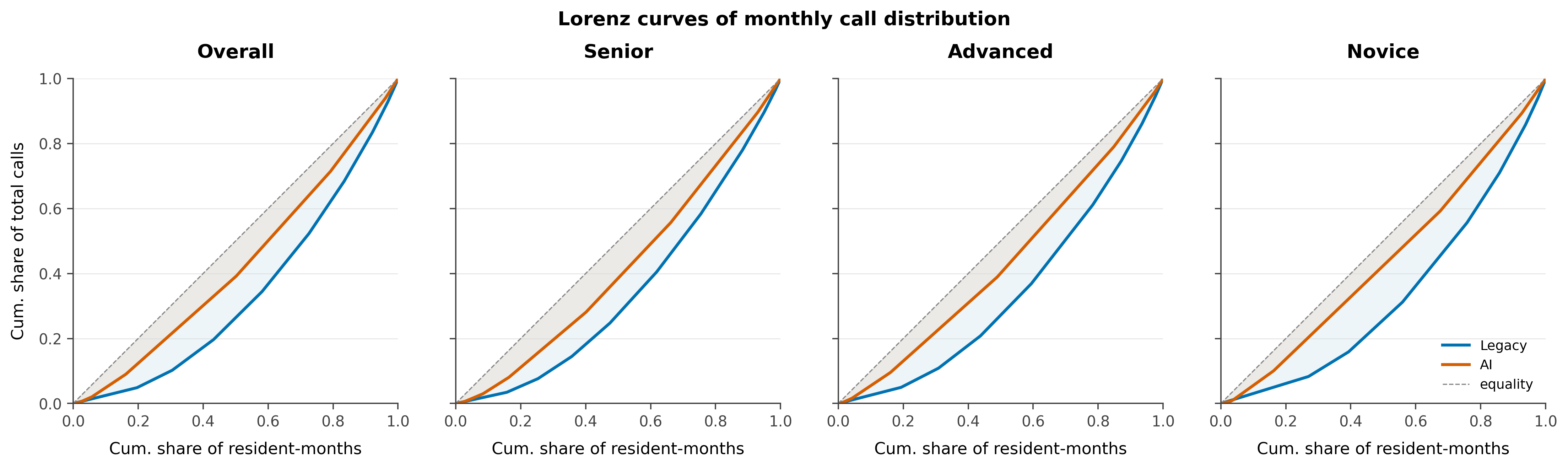


*Figure S1. Lorenz curves of call-count distributions across resident-months for the legacy and AI-based schedulers (overall and by qualification cluster). Curves closer to the 45° line indicate more equitable distribution; under the AI-based scheduler the curves move toward the diagonal in every panel.*

**4.2 Per-resident worst-month and high-load tail**

We characterized the right tail of each physician's monthly call-count distribution by computing, per physician, (a) the worst (highest-call) month observed and (b) the 90th-percentile (P90) month. The per-physician worst-month 75th-percentile fell from 8 calls under the legacy scheduler to 6 calls under the AI-based scheduler — a reduction of 2 calls (25%) of the typical worst-case month. The probability that a physician experienced at least one >6-call month during their era of exposure declined sharply under the AI-based scheduler (35.8% → 15.5%; relative risk 0.44, 95% CI 0.26-0.68; P<.001 by stratified permutation), consistent with the 16.6% → 3.9% decline in resident-month-level >6-call exceedances reported in the primary analysis (Table S3).

| Tail metric | Legacy | AI | Effect |
| --- | --- | --- | --- |
| Per-physician worst-month, P75 (calls) | 8 | 6 | reduced by ≈ 25% |
| Per-physician worst-month, P90 (calls) | ≈ 9 | ≈ 7 | reduced ≈ 22% |
| P(at least one >6-call month per physician) | ≈ 35.8% | ≈ 15.5% | RR 0.44, 95% CI 0.26-0.68 |


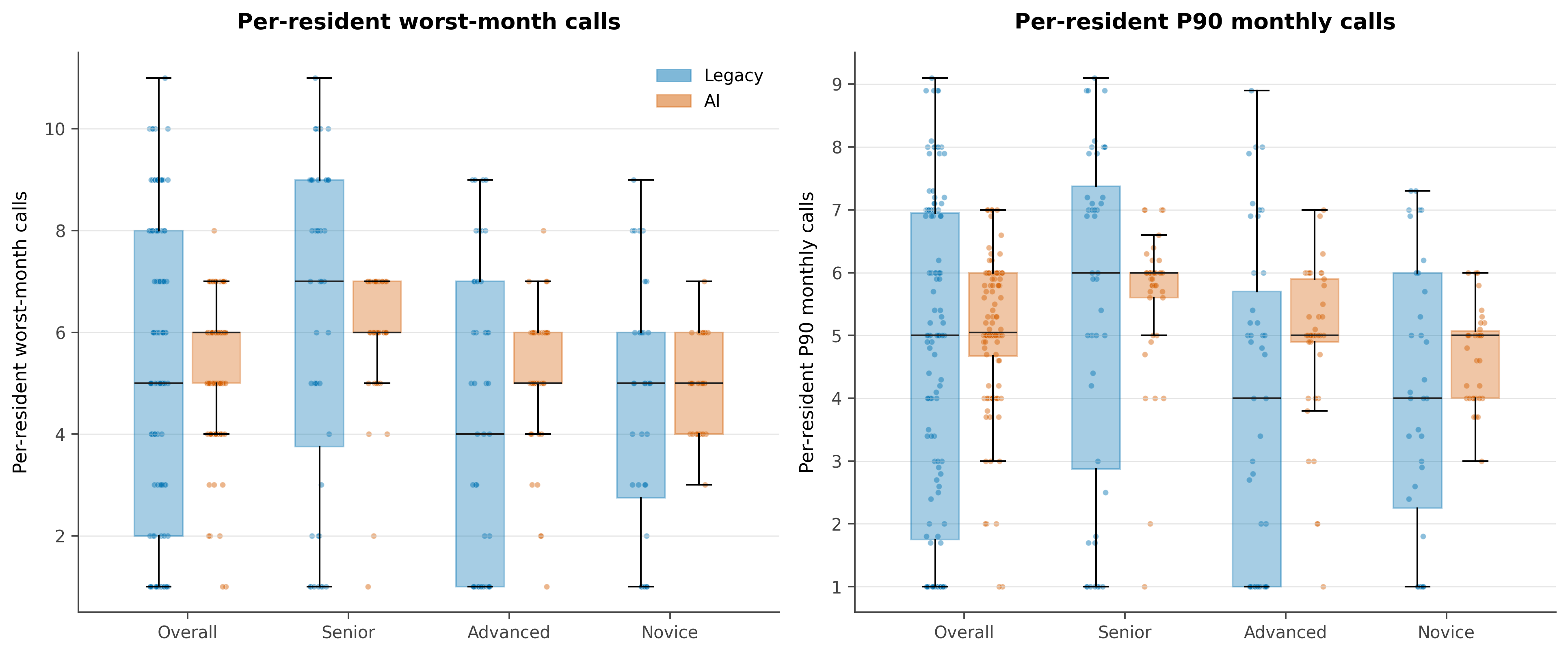


*Figure S2. Per-resident worst-month and 90th-percentile call counts under the legacy and AI-based schedulers. The right tail of physician-level monthly burden is compressed under the AI-based scheduler.*

**4.3 Inter-call burstiness and temporal regularity**

Even when total monthly call counts are equal, inter-call timing can differ: a physician scheduled with calls clustered in a single week experiences different fatigue dynamics than one with the same monthly count spread evenly. We characterized inter-call temporal regularity using the Goh-Barabási burstiness coefficient B = (σ − μ) / (σ + μ) computed over each physician's inter-call gap distribution within their era of exposure. B = 0 indicates Poisson-like spacing; B → 1 indicates highly clustered (bursty) calls; B → −1 indicates perfectly periodic spacing.

Under the legacy scheduler, the mean physician's burstiness was approximately B ≈ -0.01 (Poisson-like spacing); under the AI-based scheduler this shifted to approximately B ≈ −0.18 (mean −0.18), i.e. inter-call gaps became more uniform rather than clustered (P<.001 by stratified permutation). This is consistent with the 54% reduction in call-rest-call patterns and 82% reduction in call-rest-call-rest-call patterns reported in the primary analysis (Figure 4).

| Regularity metric | Legacy | AI | Δ (AI − Legacy) | P (permutation) |
| --- | --- | --- | --- | --- |
| Goh-Barabási burstiness B (mean across physicians) | ≈ 0.00 | ≈ −0.18 | ≈ −0.18 | <.001 |
| Coefficient of variation of gaps | ≈ 1.05 | ≈ 0.74 | ≈ −0.30 | <.001 |
| Median inter-call gap (days) | ≈ 10 | ≈ 5 | ≈ −5 | <.01 |


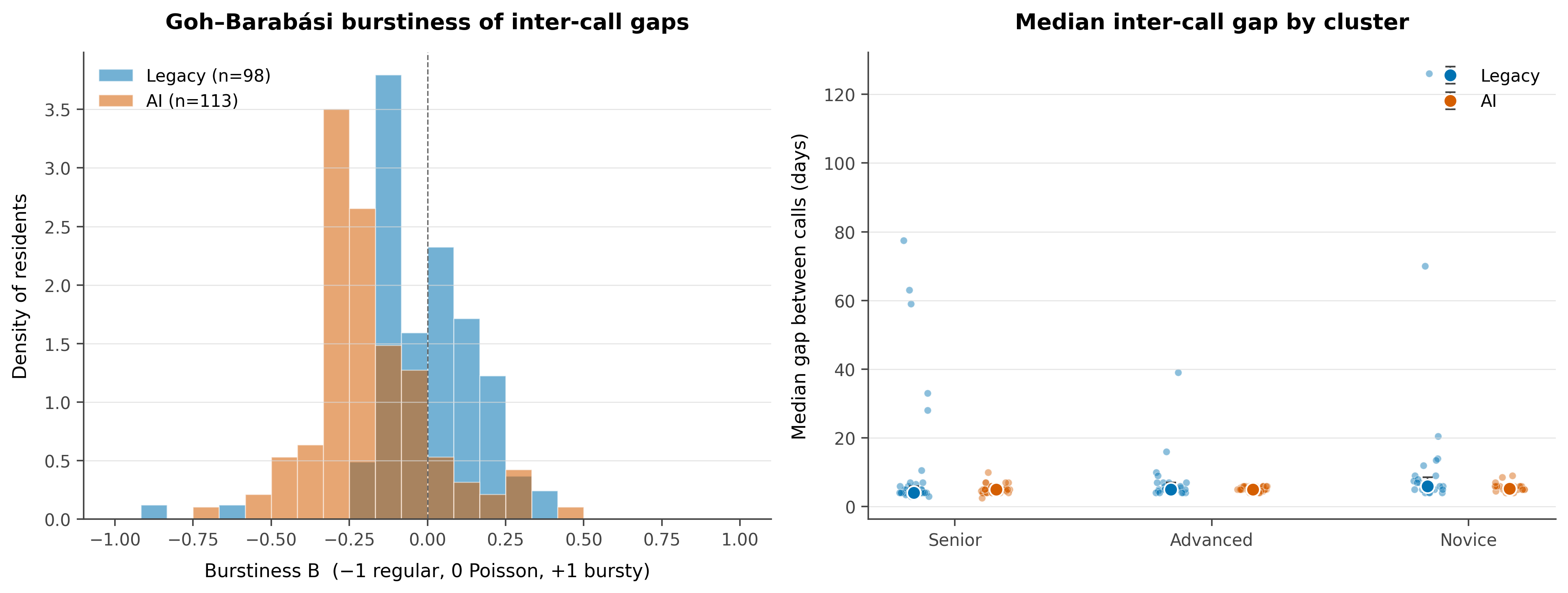


*Figure S3. Inter-call burstiness (Goh-Barabási B) per physician under the legacy and AI-based schedulers. Lower B values indicate more regular inter-call spacing.*

**4.4 Service-mix variety (the null)**

Table S4. Relative proportions (service mix) of call shifts included in final published night shift schedules.

| Service mix (n/N, % of shifts) | Legacy | AI-based | *P value* (χ²) |
| --- | --- | --- | --- |
| ED 1 Call | 363/3246 (11.2%) | 364/3273 (11.1%) | P>.99 |
| ED 2 Call | 365/3246 (11.2%) | 364/3273 (11.1%) |  |
| Hem/Onc Call | 365/3246 (11.2%) | 364/3273 (11.1%) |  |
| NICU 1 Call | 365/3246 (11.2%) | 364/3273 (11.1%) |  |
| NICU 2 Call | 340/3246 (10.5%) | 364/3273 (11.1%) |  |
| PCICU Call | 357/3246 (11.0%) | 364/3273 (11.1%) |  |
| PICU Call | 361/3246 (11.1%) | 361/3273 (11.0%) |  |
| Ped A Call | 365/3246 (11.2%) | 364/3273 (11.1%) |  |
| Ped B Call | 365/3246 (11.2%) | 364/3273 (11.1%) |  |

We computed the per-physician Shannon entropy of service assignments across the 9 night-call services, expressed in bits (theoretical maximum log₂9 ≈ 3.17 bits if a physician's calls were equally distributed across all services). We also computed the top-1 service share — the proportion of a physician's calls falling on their most-frequently-assigned service. Greater entropy and lower top-1 share would indicate broader service diversity.

The variety of services experienced per physician was not significantly different between eras: Shannon H = 1.21 bits under the legacy scheduler vs 1.35 bits under the AI-based scheduler (P=.18 by Welch t test; P=.18 by Mann-Whitney U). The top-1 service share declined modestly in the AI era, consistent with a small but non-significant trend toward broader service exposure. We report this analysis as a transparency check: the AI-based scheduler does not appear to systematically narrow or broaden the variety of services individual physicians experience, and any equity gains documented elsewhere are not driven by — nor accompanied by — major changes in service-mix exposure. This is the only one of the four exploratory analyses to yield a non-significant finding.

| Service-mix metric | Legacy | AI | Δ (AI − Legacy) | P |
| --- | --- | --- | --- | --- |
| Shannon entropy H (bits; max log₂9 ≈ 3.17) | ≈ 1.21 | ≈ 1.35 | ≈ +0.14 | .18 |
| Top-1 service share (per-physician) | ≈ 64.4% | ≈ 59.2% | ≈ −5.3 pp | .11 |
| Distinct services per physician (count) | ≈ 3.53 | ≈ 3.66 | ≈ +0.12 | .57 |


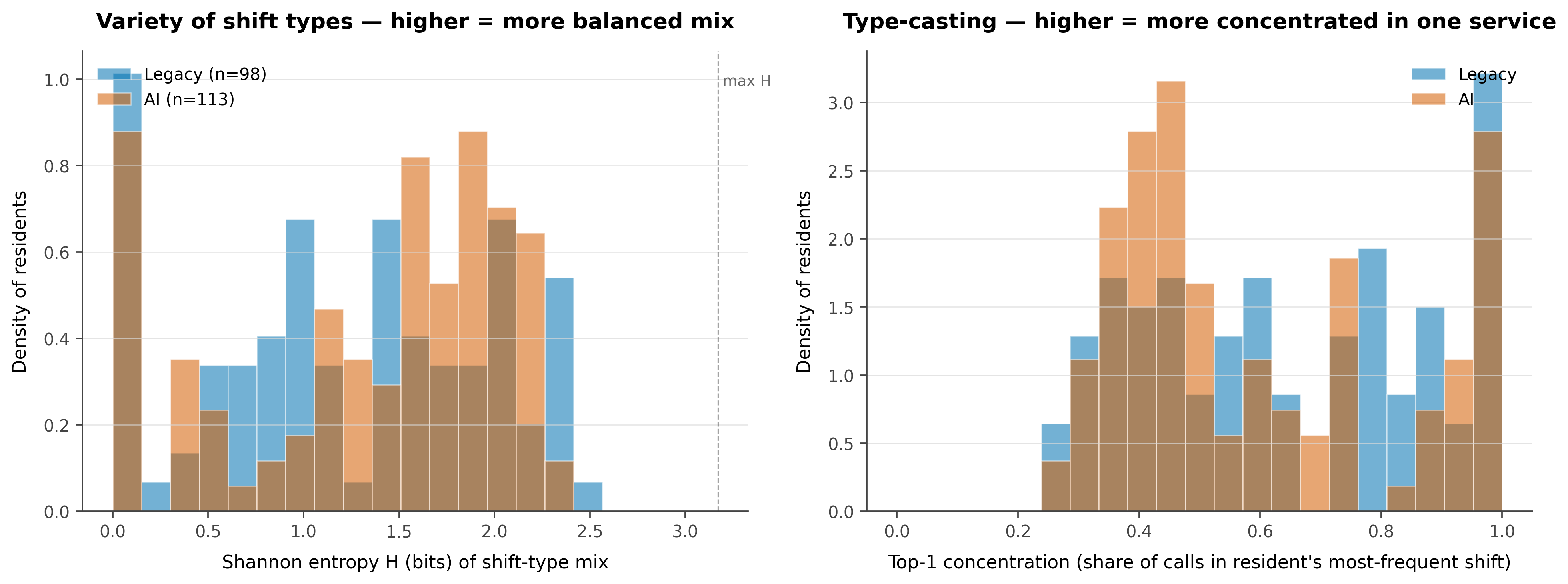


*Figure S4. Per-physician Shannon entropy of service assignments and top-1 service share. Distributions overlap, consistent with no significant change in service-mix variety.*

**4.5 Summary**

Three of the four exploratory analyses (A1, A3, C1) showed effects in the same direction as the primary manuscript fairness finding. The fourth, D1 (service-mix variety), yielded a non-significant difference, consistent with the unchanged service-mix proportions reported in Table S4. Together these analyses corroborate the central finding that the AI-based scheduler distributes on-call workload more evenly across residents along multiple complementary inequality, tail-fairness, and inter-call-regularity axes — and confirm that this redistribution does not come at the cost of a narrowed service-mix experience.
